# Virtual clinical trials via a QSP immuno-oncology model to simulate the response to a conditionally activated PD-L1 targeting antibody in NSCLC

**DOI:** 10.1007/s10928-024-09928-5

**Published:** 2024-06-10

**Authors:** Alberto Ippolito, Hanwen Wang, Yu Zhang, Vahideh Vakil, Aleksander S. Popel

**Affiliations:** 1 Department of Biomedical Engineering, Johns Hopkins University School of Medicine, Baltimore, MD 21205 USA; 2 Clinical and Quantitative Pharmacology, CytomX Therapeutics, Inc., South San Francisco, CA USA; 3 Department of Oncology, and the Sidney Kimmel Comprehensive Cancer Center, Johns Hopkins University School of Medicine, Baltimore, MD 21205 USA

**Keywords:** PROBODY® Activatable therapeutics, Pharmacology, Immunology, Oncology, Virtual patients, Omics, Quantitative systems Pharmacology

## Abstract

Recently, immunotherapies for antitumoral response have adopted conditionally activated molecules with the objective of reducing systemic toxicity. Amongst these are conditionally activated antibodies, such as PROBODY® activatable therapeutics (Pb-Tx), engineered to be proteolytically activated by proteases found locally in the tumor microenvironment (TME). These PROBODY® therapeutics molecules have shown potential as PD-L1 checkpoint inhibitors in several cancer types, including both effectiveness and locality of action of the molecule as shown by several clinical trials and imaging studies. Here, we perform an exploratory study using our recently published quantitative systems pharmacology model, previously validated for triple-negative breast cancer (TNBC), to computationally predict the effectiveness and targeting specificity of a PROBODY® therapeutics drug compared to the non-modified antibody. We begin with the analysis of anti-PD-L1 immunotherapy in non-small cell lung cancer (NSCLC). As a first contribution, we have improved previous virtual patient selection methods using the omics data provided by the iAtlas database portal compared to methods previously published in literature. Furthermore, our results suggest that masking an antibody maintains its efficacy while improving the localization of active therapeutic in the TME. Additionally, we generalize the model by evaluating the dependence of the response to the tumor mutational burden, independently of cancer type, as well as to other key biomarkers, such as CD8/Treg Tcell and M1/M2 macrophage ratio. While our results are obtained from simulations on NSCLC, our findings are generalizable to other cancer types and suggest that an effective and highly selective conditionally activated PROBODY® therapeutics molecule is a feasible option.

**Supplementary Information:**

The online version contains supplementary material available at 10.1007/s10928-024-09928-5.

## Introduction

In recent years, the PROBODY® therapeutics (Pb-Tx) approach has emerged as a potential strategy for cancer immunotherapy [1]. This drug design is part of a modern protocol of conditional activation of drugs in the tumor microenvironment (TME). Essentially, these approaches exploit the unique properties of the TME to selectively activate the compound in the presence of target cancer cells. Specifically, Pb-Tx molecules are designed to respond to the tumor-resident proteases. The Pb-Tx design consists of three components: the original molecule, a masking peptide and a substrate bridge that links the former two components [2]. In the case of checkpoint inhibitors, the original antibody is connected via the bridges to two masks, which can reversibly reveal the molecule’s active sites. The linker, on the other hand, maintains the peptide mask close to the active site and it is cleavable by proteases that are active in the TME, such as tumor-associated serine protease matriptase (MT-SP1) and urokinase plasminogen activator (uPA) [3]. Thus, these proteases digest the substrate bridge in the TME, ultimately increasing the specificity of the molecule’s activity on tumor cells.

In parallel to the clinical trial, quantitative systems pharmacology (QSP) models have been developed to describe and predict the kinetics of Pb-Tx molecules [4–6]. Previously, a Quantitative Systems Pharmacology for Immuno-oncology (QSP-IO) model was developed to predict the response of anti-PD-L1 checkpoint inhibitors with the Pb-Tx design versus the original molecule [6]. In [6], they considered virtual clinical trials on triple negative breast cancer (TNBC) and determined that the Pb-Tx therapy would be predicted to be slightly less effective but more localized to the tumor compared to the standard antibody [7]. The predictions in [6] showed a similar response rate to Phase 1 clinical trial data [8]. However, the virtual patient (VP) selection was based on a previously published method [6, 9] where the patient parameters were sampled from distributions calibrated on clinical, in-vivo, and in-vitro data. While this method is common, the field is moving towards selecting VPs closer to real patients, thus the selection criteria should be based on clinical observables such as immunogenomic biomarkers.

In the present study, we extend the QSP model of TNBC to investigate activation of anti-tumor immune response in non-small cell lung cancer (NSCLC) during Pb-Tx therapy. We deploy the previously proposed framework [6], calibrate the Pb-Tx molecule to NSCLC and then run anti-PD-L1 monotherapy in virtual clinical trials. Unlike the previous work [6], we have chosen to select VPs using real patient immune cell ratios, similarly to a recent study [10]. In fact, we construct an inclusion probability using the omics data from the iAtlas database [11] and from our simulations to select realistic VPs.

Our results suggest that masking the PD-L1 targeting antibody in NSCLC has negligible improvement on the efficacy, while improving the localization of active therapeutic molecule in the TME. We also perform a parameter sensitivity analysis and biomarker analysis to suggest key observables that control the response rate of virtual patients to the Pb-Tx. While we have extended these results to NSCLC, we also extend the study to a pan-cancer setting by analyzing the behavior of the overall response rate (ORR) as a function of the tumor mutational burden (TMB).

## Materials and methods

### Virtual patient selection

An accurate selection of virtual patients is essential to ensure the reproducibility, accuracy, and breadth of information that a virtual clinical trial can provide. In practice, each virtual patient represents a set of unique input parameters and initial conditions used to simulate the model. These are usually determined via sampling from calibrated (or assumed) distributions or are obtained from pre-treatment simulations. Ideally a substantial virtual patient cohort is capable of representing both individual patients and population response.

**Fig. 1:**
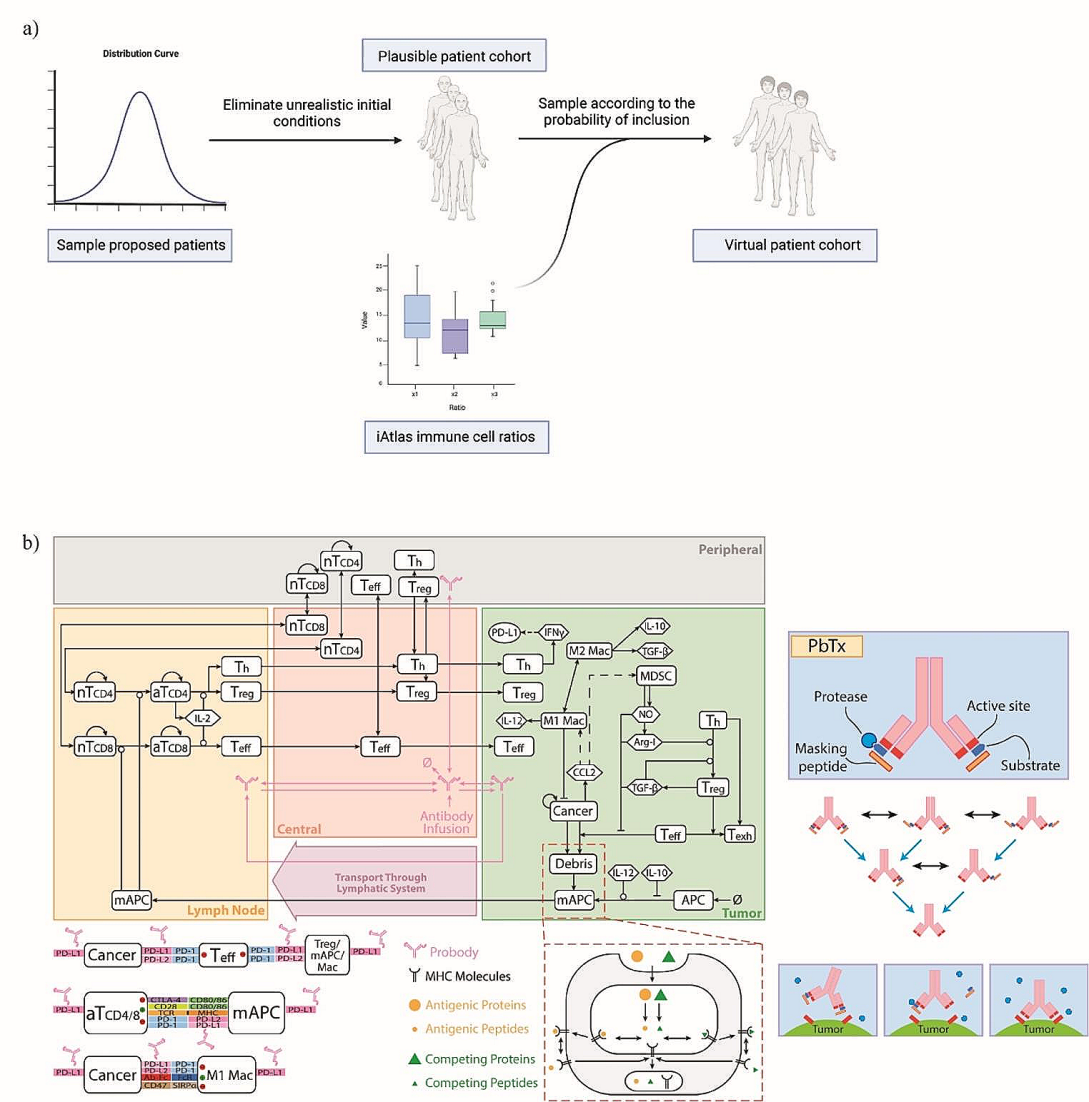
(**a**) Algorithm for virtual patient selection based on pre-treatment immune subset ratios derived from iAtlas omics database. (**b**) Quantitative Systems Pharmacology model for checkpoint inhibition using Pb-Tx. Natural death/degradation of cellular/molecular components was omitted from the figure. The figure also includes a cartoon showing the Pb-Tx unmasking, cleaving and binding

**Fig. 2:**
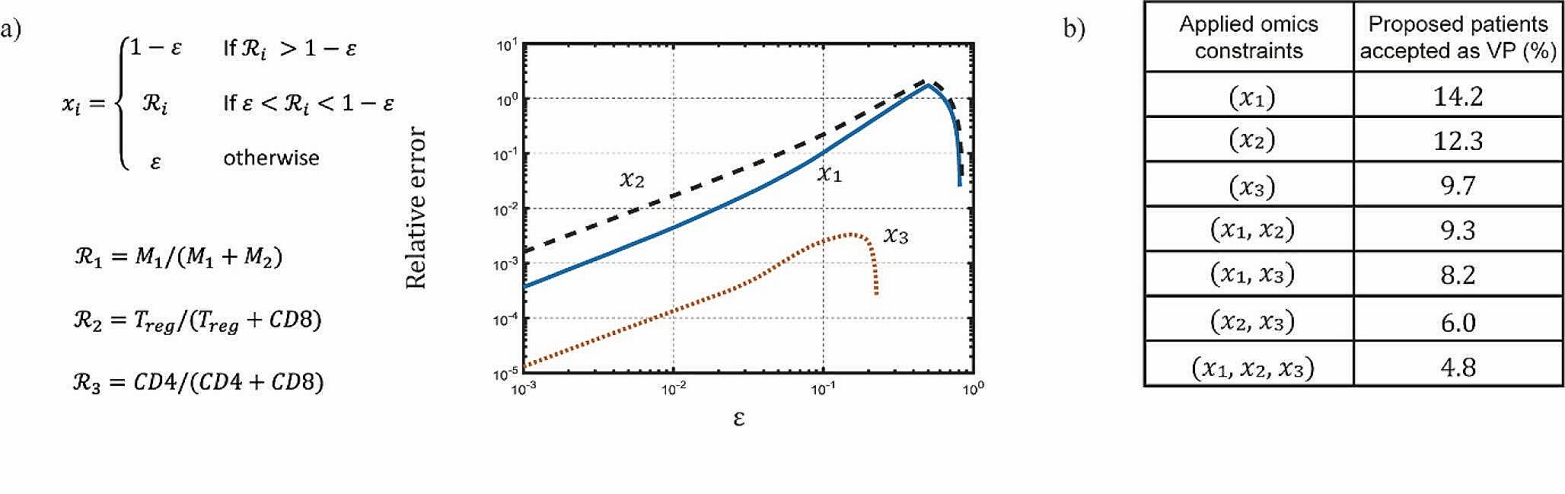
(**a**) Definition of the immune ratios ${x}_{1}$, ${x}_{2}$ and ${x}_{3}$. The relative error in the mean distribution of the ratios is shown as a function of $\epsilon$. (**b**) Acceptance rate percentage of the proposed patients as virtual patients for each combination of immune-omics constraints

In this work, we implement the virtual patient selection method described in Fig. 1a (see Supplementary information for additional details). This method is adapted from [10, 12]. As the first step, the input parameters are randomly selected from estimated distributions via Latin Hypercube Sampling [13, 14]. We designate these patients as “proposed patients” and proceed by simulating their tumor growth from a few cells for 8000 days, using the QSP-IO model described in Fig. 1b; note that the 8000 day interval is a computational parameter and does not necessarily correspond to the real tumor growth duration. We also want to emphasize that this initial condition calculation is of negligible complexity compared to the virtual clinical trial simulation. We then select the patients that have plausible tumor size profiles, and we designate them as “plausible patients.” The initial condition (i.e., pre-treatment condition), in terms of all the cell and cytokine densities, of the plausible patients is set as the timepoint when the tumor reaches the desired initial tumor diameter (i.e., pre-treatment tumor size). Using these initial conditions, we construct three immune subset ratios shown in Fig. 2a and defined as:

1a $${x_1} = \left\{ {\begin{array}{*{20}{c}}{1 - \varepsilon }&{if\;{M_1}/\left( {{M_1} + {M_2}} \right) > 1 - \varepsilon } \\ {\frac{{{M_1}}}{{{M_1} + {M_2}}}}&{\varepsilon \leqslant {M_1}/\left( {{M_1} + {M_2}} \right) \leqslant 1 - \varepsilon } \\ \varepsilon &{{M_1}/\left( {{M_1} + {M_2}} \right) < \varepsilon } \end{array}} \right.$$

1b $${x_2} = \left\{ {\begin{array}{*{20}{c}}{1 - \varepsilon }&{if\;{T_{reg}}/\left( {{T_{reg}} + CD8} \right) > 1 - \varepsilon } \\ {\frac{{{T_{reg}}}}{{{T_{reg}} + CD8}}}&{\varepsilon \leqslant {T_{reg}}/\left( {{T_{reg}} + CD8} \right) \leqslant 1 - \varepsilon } \\ \varepsilon &{\frac{{{T_{reg}}}}{{{T_{reg}} + CD8}} < \varepsilon } \end{array}} \right.$$

1c $${x_3} = \left\{ {\begin{array}{*{20}{c}}{1 - \varepsilon }&{if\;CD4/\left( {CD4 + CD8} \right) > 1 - \varepsilon } \\ {\frac{{CD4}}{{CD4 + CD8}}}&{\varepsilon \leqslant CD4/\left( {CD4 + CD8} \right) \leqslant 1 - \varepsilon } \\ \varepsilon &{CD4/\left( {CD4 + CD8} \right) < \varepsilon } \end{array}} \right.$$

Here, ${M}_{1}, {M}_{2}, {T}_{reg}, CD8$ and $CD4$ are the tumor densities of the M1 and M2 macrophages, and regulatory, CD8 and CD4 T cells. These immune subset ratios are a modification of the ones presented by [10]. In particular, we add a new parameter $\epsilon$, which is a positive, and less than 1, divergence-avoiding parameter. With this definition, we are able to avoid any singularities associated with very small or zero denominators when we consider simple ratios of cell numbers. We then compare the ratios in plausible patients with the same immune subset ratios constructed using the omics data from the iAtlas database. The comparison follows the procedure set by [12]. Similarly to [10], we adopt the ratios for comparison because the iAtlas data (i.e., proportions of immune subsets in leukocytes) do not directly correspond to the unit of cell densities in our model (cells/mL tumor). Thus, we estimate the probability density of the immune subset ratios based on their distributions in iAtlas data and plausible patients. Then, we define the probability of inclusion as the ratio of probability density between iAtlas data and plausible patients. In front of this ratio is a normalization factor $\beta$, which is obtained via simulated annealing [12]. Using this probability of inclusion, we proceed to select the virtual patients from our plausible patient cohort. A detailed algorithm can be found in the Supplementary information (Fig. S1). Finally, this virtual patient cohort is used to conduct virtual clinical trials. While the iAtlas database contains a plethora of omics data of many kinds, we have selected to use the three immune subset ratios since the involved immune cells are explicitly modeled in our framework.

### Overview of the QSP-IO model informed with PbTx dynamics

QSP-IO models are systems biology type frameworks used to predict population and individual responses to immuno-, chemo- and combination therapies. Usually, the systems in question are divided into multiple compartments, each representing a different tissue or organ or their assemblies, inside which a set of explicitly modeled molecular or cellular species can interact or move between compartments. In particular, QSP-IO models are informed with the PKPD relations governing the drug as well as the interactions between the immune system and tumor cells. Each simulation is run for a different virtual patient which is the set of parameters describing the mechanistic relations or properties of each species and compartment.

In this work, we implement the previously proposed QSP-IO model for the evaluation of pacmilimab monotherapy, a PbTx molecule, as the anti-PD-L1 immune checkpoint inhibitor [6]. While we provide a detailed explanation of the theory and implementation in the Supplementary information, here we give a brief overview of the model.

The model used in our work, shown in Fig. 1b, contains 4 distinct compartments: the tumor, tumor-draining lymph node, peripheral, and central compartment. The tumor compartment contains the cancer and stromal cells and is the location where the tumor interacts with the components of the immune system in the TME. The tumor-draining lymph nodes are the location where antigen presentation and T cell maturation occur. The peripheral compartment describes the rest of the body and it is generally used to describe unwanted activity of the therapy. Finally, the central compartment represents blood flow which connects all the compartments.

The model contains the dynamics of several different cell types from the immune system as well as those of the tumor. These dynamics are divided into 11 different modules, each describing different cells or key molecular species interactions. Within these modules, we explicitly model the maturation, activation, depletion, and death of CD8, CD4 and regulatory (Treg) T cells. These cells come into contact with mature antigen presenting cells (APCs) arriving from the TME. These APCs mature due to the release of cytokines in the TME. Additionally, macrophages in the M1 and M2 configurations are also modeled in the TME. The interactions between these different cells are ascribed to the immune synapse compartments. This compartment includes all the different ligands used for immune-regulatory actions, such as PD-L1, PD-1, PD-L2, CTLA4, CD28, TCR, MHC and CD80, in the synapse. Of the 11 modules, 10 were previously implemented by Wang et al. [9] and the additional compartment to explicitly model the out-of-synapse dynamics was introduced by Ippolito et al. [6]. We explicitly include only PD-L1 in the out-of-synapse compartment, since it is the only ligand that interacts with the Pb-Tx in this study. However, we want to emphasize that the same procedure can be applied to the other ligands, i.e. CTLA4 and PD-1 and we still explicitly model the interactions between PD-L1, PD-1, PD-L2, CTLA4, CD28, TCR, MHC and CD80 in the synaptic compartment as these are immune-regulatory.

Pacmilimab is an antibody made into a Pb-Tx by the addition of a mask covering the active sites and kept in proximity thereto by a cleavable substrate linker. The PK model describing the Pb-Tx dynamics has been described previously by Stroh et al. [4, 5]. Briefly, the mask is allowed to stochastically and reversibly shift and reveal the active site. This unmasking rate is governed by an equilibrium constant ${K}_{M}$, which is the likelihood that the mask will reveal the active site. If this were the only addition to the antibody, then the Pb-Tx would just be a less active form of its parent solely due to the reduced exposure of the active site. However, this reduced exposure is made selective since the key property of the Pb-Tx is that the substrate holding the mask is cleavable by proteases that are active in the TME. These proteases are tumor-associated serine protease matriptase, urokinase plasminogen activator and cysteine protease legumain [1] for pacmilimab; however, it is possible to select other substrates that are digestible by different enzymes [15, 16]. We calibrate the model using previously published model parameters from [10], such as the tumor growth rate [17], with the addition of the Pb-Tx specific parameters from Ippolito et al. [6] calibrated to NSCLC by modifying the ${k}_{cvg}$ to represent the tumor protease activity. In particular, we have shifted the median of the patient distribution of ${k}_{cvg}$ according to the expressions of the enzymes in [15, 16] and we have maintained the same variation interval described in [6]. All species, chemical reactions, ordinary differential equations governing the processes, and model parameters are provided in the Supplementary information files.

### Calibration of the immune subset ratios

In the previous section we described the virtual patient selection criteria using omics-informed immune cell ratios. Here we will describe the calibration of these ratios and the selectivity of the probability of the inclusion imposed by each immune ratio or constraint.

The probability of inclusion is built based on the log of the immune ratios [10], thus it is subject to both positive and negative divergences, i.e. plus and minus infinity. Compared to previous work [10], the positive divergences are avoided by defining the constraints as immune cell ratios instead of fraction. On the other hand, the negative divergence is avoided by introducing the constant $\epsilon$ which bounds the maximum and minimum value of the ratio between $1-\epsilon$ and $\epsilon$, as shown in Fig. 2a. We proceed evaluating the relative error of the mean, shown in Fig. 2a, defined as $({\stackrel{-}{y}}_{i} \left({\upepsilon }\right)-{\stackrel{-}{y}}_{i})/{\stackrel{-}{y}}_{i}$, where ${\stackrel{-}{y}}_{i} \left({\upepsilon }\right)$ is the median of the iAtlas immune fraction distribution modified by $\epsilon$ and and ${\stackrel{-}{y}}_{i}$ is the median of the original iAtlas distribution. The dips in the plot correspond to when $\epsilon$ is equal to the median of the distribution of the given ratio ${x}_{i}$ (see Fig. S2 for distribution comparisons).

Once the ratios ${x}_{i}$ were defined, we analyze how constraining or how low they reduce the acceptance rate of virtual patient selection from the plausible patient cohort. We summarize the results in the table shown in Fig. 2b, where we begin from the same cohorts of proposed patients (and plausible patients) and we then calculate the probability of inclusion and select the virtual patients from the initial set. We report the percentage of initial proposed patients finally accepted as virtual. We noticed that, when applying only a single ratio as the constraint, the ${x}_{3}$ was the one that allowed for the lowest percent compared to the other 2 ratios (9.7 vs. 14.2 and 12.3 for ${x}_{1}$ and ${x}_{2}$ respectively). We assume that this may be due to the property of the model, since CD4 cells include a spectrum of cell types which we may not be representing fully. We note that amongst the CD4 cells we explicitly model both Tregs and T helper cells. Additionally, by applying more constraints simultaneously the final acceptance rate is lower than the acceptance rate of either constraint applied singularly. This observation is reasonable since applying more constraints is expected to make a more stringent selection rate for VPs from plausible patients.

## Results

### Pb-Tx anti-PD-L1 monotherapy applied to NSCLC

Now that we have calibrated the QSP-IO model with the Pb-Tx molecule dynamics [6], we proceed to evaluating the therapy in terms of effectiveness. Since there is no reported clinical trial of Pb-Tx therapy for NSCLC, we imagine doing an exploratory prospective study using the same dosing schedule as for TNBC reported in [6]. The proposed dosing regimen based on the trial is 10 mg/kg every 2 weeks (Q2W) for a duration of 400 days, which is similar to other PD-L1 immune-checkpoint inhibitor therapies [18]. We proceed by simulating two cases for the same *N* = 20,000 proposed patients resulting in 460 virtual patients: unmasked antibody monotherapy and Pb-Tx monotherapy. The “unmasked antibody” monotherapy refers to injecting an antibody without any masks, i.e., atezolizumab. Since the unmasked antibody monotherapy will serve as the reference case, we assume that it will also be applied with the same dosing schedule as pacmilimab, i.e., 10 mg/kg administered Q2W. See Fig. S3 for PK of each Pb-Tx state.

**Fig. 3:**
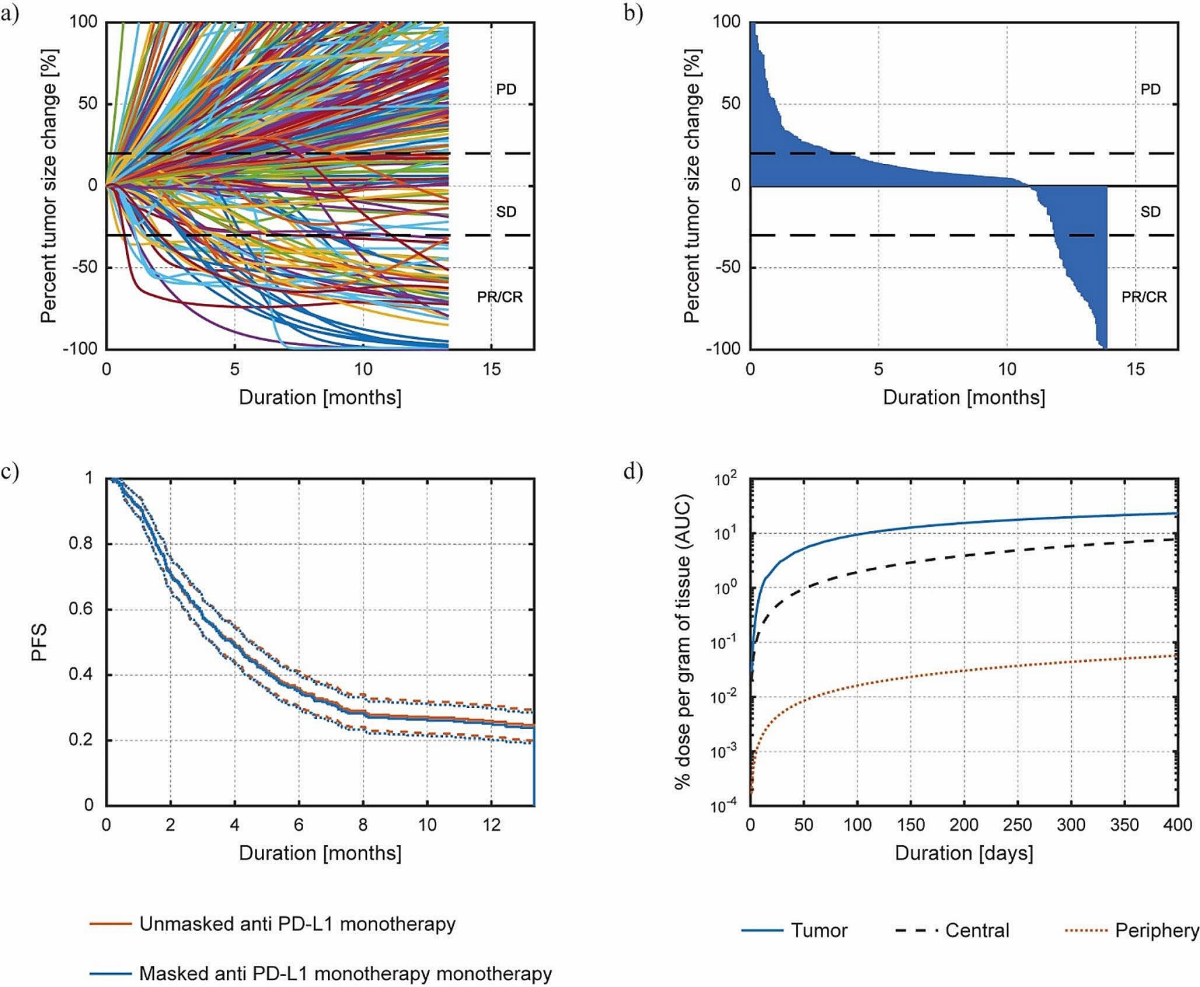
(**a**) Spider plot showing the change in tumor size of the virtual patient cohort. (**b**) Waterfall plot showing the post-treatment results from simulated clinical trials on NSCLC for Pb-Tx monotherapy. (**c**) Progression free survival of masked vs. unmasked monotherapy shown as the mean (continuous line) surrounded by 95% confidence intervals (dotted lines). (**d**) Area under the curve of the percent dose per gram of tissue for each compartment (tumor, central and periphery) of the Pb-Tx monotherapy

In Fig. 3a, we show the spider plots of the virtual patient cohort in Pb-Tx monotherapy. We also trace dotted limits at 20% and − 30% following the RECIST 1.1 criteria [19]. Specifically, we label the regions of progressive disease (PD), stable disease (SD) and partial/complete response (PR/CR). The spider plot shows mostly monotonic tumor size change as patient response (typical for anti-PD-L1 therapies [9]). To better quantify patient response, we build a waterfall plot based on the final day of the simulated clinical trial in Fig. 3b. As with the spider plots, we also trace the dotted lines following the RECIST 1.1 criteria. We find that the response is around 16%, which is similar to the response reported for the original anti-PD-L1 therapy of 18%. To further solidify this similarity, we plot the progression free survival (PFS), or the length of time during the treatment where the tumor does not progress, of the Pb-Tx vs. original anti-PD-L1 therapy in Fig. 3c. We show in this plot the two solid curves with the dotted lines that represent the 95% confidence intervals (CI). Clearly, the curves are almost indistinguishable and well within the CI, thus, even though the response to the masked therapy looks slightly lower, the response to these therapies is statistically similar.

The strength of the Pb-Tx therapy lies in the ability of the molecule to become selectively active in the TME. Previously, Ippolito et al. [6] had predicted an increase in the localization of the active molecules in the tumor for the Pb-Tx therapy compared to the rest of the body for TNBC. Here, we extend this observation to NSCLC by measuring the exposure to the active molecule by using the % dose per gram of tissue. We define as active molecules any Pb-Tx molecule which has at least one active site revealed at any point in time, whether it is by unmasking or cleaving. We then evaluate the area under the curve (AUC) of the %dose per gram of tissue and we report it in Fig. 3d. The results predict a much higher exposure in the tumor, at least 1 order of magnitude, compared to the other compartments. Interestingly, the exposure in the peripheral compartment is always at least two orders of magnitude lower than the rest. This suggest that the periphery is relatively protected from the drug while the tumor is exposed to the bulk of the therapeutic action.

### Sensitivity analysis and key biomarkers in patient response

We have discussed the efficacy and localization of the Pb-Tx monotherapy. We now proceed to discuss the key parameters or physical mechanisms governing the simulated patient response to pacmilimab.

**Fig. 4:**
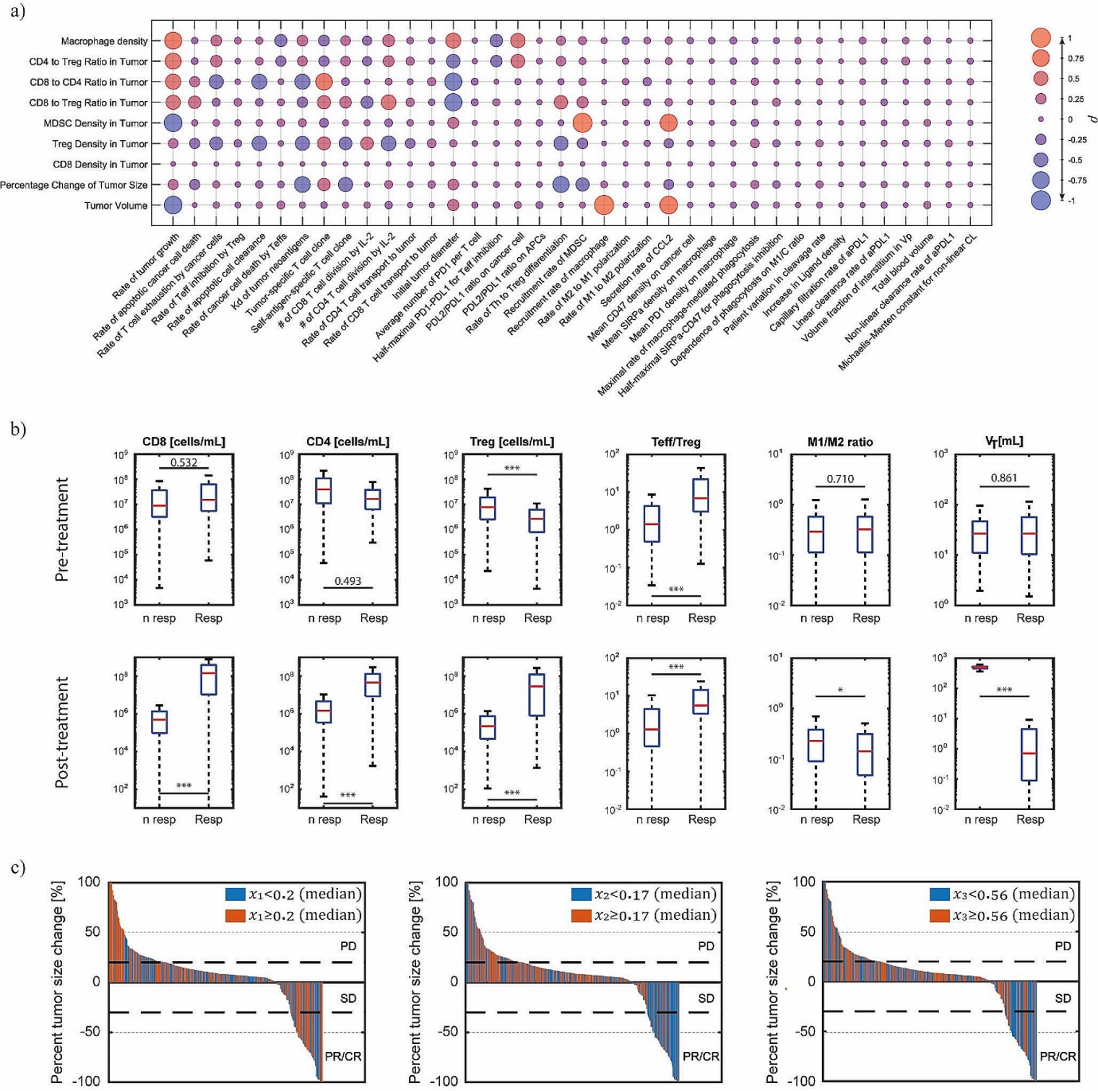
(**a**) Sensitivity analysis via PRCC of the virtual patient parameters (x-axis) versus the model biomarkers (y-axis). (**b**) Boxplots of key biomarkers pre- and post-treatment for responders and non-responders of Pb-Tx monotherapy for NSCLC. The p-values are indicated and the stars *, **, *** indicate a p-value < 0.05, 0.01 and 0.001 respectively. (**c**) Waterfall plots where each VP is color-coded based on whether its immune ratio is above (red) or below (blue) the median value

As a first step we ran a sensitivity analysis via partial rank correlation coefficient (PRCC) [20] and report the results as a bubble plot in Fig. 4a. Here, we study the correlation between the dependence of some key biomarkers, such as tumor size and immune cell densities, as a function of the patient dependent variables to determine which variable may govern response. For each biomarker, we plot the correlation coefficients, $\rho$, with the VP parameters as bubbles. The size of the bubble represents the magnitude of the correlation coefficient. The higher the absolute value, the larger the bubble. The second piece of information that the bubbles provide is the color: closer to red means positive correlation while close to blue is equivalent to negative correlation. For example, the sensitivity analysis shows how increasing T cell exhaustion by cancer cells diminishes the CD8 to CD4 ratio. Besides the rate of tumor growth and initial tumor diameter, the Kd of neoantigens and the Tumor-specific T cell clones are the most influential parameters. The Kd, the equilibrium binding constant, controls the likelihood of the ICI to block the PD-L1 (higher Kd means higher off rate). For this reason, higher Kd is associated with higher immune cell regulation seen by decrease in CD8/CD4 ratio for example. On the other hand, an increase in the tumor-specific T cell clones (TCC) are correlated with a better response. In our model, the TCC is an indirect measure of the tumor mutational burden, which we will argue in the next section will allow us to extend the predictions to a more “pan-cancer” type simulation. Another interesting observation is that the system is relatively insensitive to the change in cleavage rate. This result is expected since the cleavage rate is relatively slow compared to the other timescales at play and especially for high enough doses where the reversible unmasking, while small, still suffices to allow the ICI to block most of the receptor.

In addition to the sensitivity analysis, we assessed the difference in distinct biomarkers between simulated responders and non-responders. In particular, we assessed the CD8 (or Teff), CD4, Treg densities, Teff/Treg and M1/M2 ratios under pre-treatment and post-treatment conditions, in Fig. 4b. From these panels, we observe that T cell densities are higher in responders compared to non-responders. Additionally, the Teff/Treg ratio is higher in responders. Finally, it appears that the M1/M2 ratio is higher in non-responders, a characteristic of the model explained by Wang et al. [9]. Briefly, while M2 macrophages exhibit immunosuppressive activities, there is a correlation between M1-like macrophages and immunosuppressive species, which was also observed clinically [9, 21]. However, as mentioned in Wang et al. [9], the role of tumor-associated macrophages in immunotherapies needs to be better understood [22]. Additionally, we report the same waterfall plot shown in Fig. 3b in Fig. 4c where each VP is color-coded based on whether the value of the immune ratio ${x}_{i}$ is greater than or equal to (red) or less than (blue) the median value. These plots suggest that high ${x}_{1}$ usually correlate with a lower likelihood of response, since most of the tumors that have increased in size are colored red. This corroborates the data from the previous plot Fig. 4b, because a high M1/M2 ratio is more likely in non- responders and the fraction ${x}_{1}$ is a derivation of this ratio. On the other hand, a low ${x}_{2}$ fraction usually correlates to higher response likelihood. This is consistent since high ${x}_{2}$ means a large proportion of Tregs compared to CD8 T cells, which is usually found in non-responders [23]. A similar result, but less striking, is observed for ${x}_{3}$, since it represents the fraction of CD4 cells to CD8 T cells.

### Tumor mutational burden as a pan-cancer response marker

We have extended the previously published virtual clinical trials from TNBC to NSCLC and have discussed the change in efficacy, localization as well as the key biomarkers in the response. While the equations governing the two tumor models are relatively similar, the parameter distributions are the ones that really differentiate the cancer modules, where 17 parameters out of 255 have been recalibrated to be cancer specific. For these reasons, even with the same Pb-Tx therapy, the objective response rate of TNBC was around 6% [6] while here for NSCLC it was closer to 16%. Amongst the parameters that differ between the models, one key parameter is the number of T cell clones. This biomarker is an indirect measure of the TMB in our model; it has been shown that TCC correlates with TMB [24]. Studies have pointed out how TMB, or a form of the TMB called a persistent tumor mutational burden (pTMB) [25], could potentially be a marker for the response to immunotherapy; especially in the case of NSCLC clonal pTMB has been shown to be the most significant TMB type to correlate with response [25]. In particular, Yarchoan et al. [26] have shown that an increase in the mutations usually correlates with an increase in the response with the therapy.

**Fig. 5:**
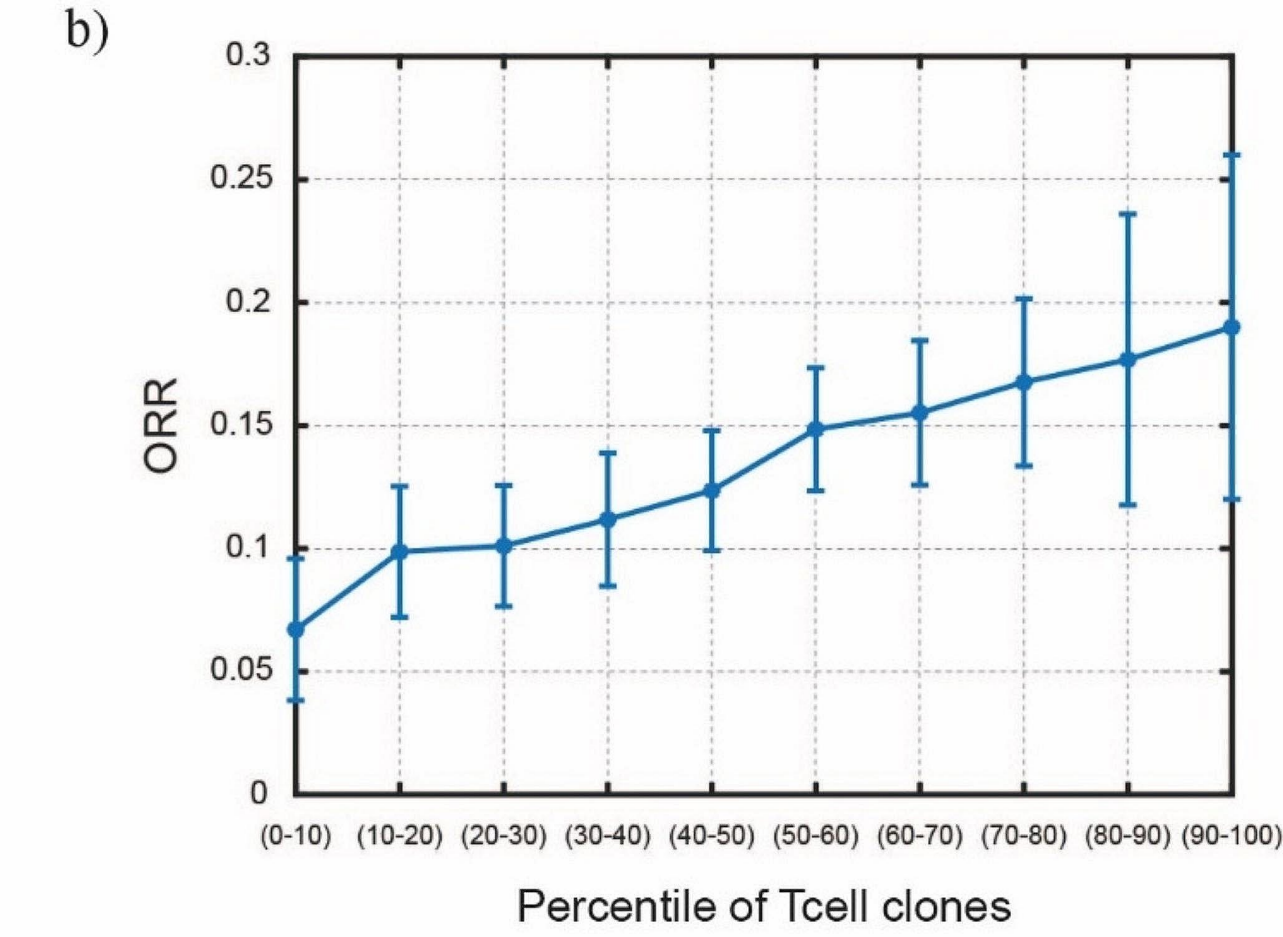
ORR calculated by the model as a function of the percentile from the distribution of T-cell clones, which is an indirect measure of the tumor mutational burden. Error bars show the 95% CI

Since the TCC spans a large interval, to test whether we can obtain a similar result as in [26], we divide the entire distribution into intervals of 10% of the total area. We construct 10 proposed patient cohorts, each of 20,000 patients, that are identical with the only difference being the TCC. In particular, group 1 will only sample the TCC which is within the first 10% of the original distribution, group 2 between 10 and 20% and so on. We report the results in Fig. 5 that suggest that an increase in the TCC is clearly correlated to an increase in the ORR. It should be noted that these results are generated for variable TCC, while otherwise keeping the simulations specific to NSCLC. Thus, even though the range of TCC may reflect the changes across different cancer types, the range of other parameters may limit the interpretation of these results as truly pan-cancer. However, qualitatively, we can compare the TCC distribution of the virtual population cohorts from our previous TNBC model [6] with the corresponding distribution in this NSCLC model. The geometric median TCC for TNBC (63) is lower than NSCLC (93) and the corresponding response rates for the same conditional anti-PDL1 therapy are 6% and 16%, therefore higher TCC correlates with better response.

## Discussion

In this paper, we have extended a QSP-IO model to study the effects of pacmilimab, a conditionally activated PD-L1 blocking antibody, applied to NSCLC. Additionally, we have introduced and calibrated a VP selection method which, using pre-treatment data from iAtlas, allowed us to select more realistic virtual patients for our virtual clinical trials. While the method for VP selection is an improvement in principle, the upside should be confirmed with more and richer datasets. We have used the model to compare the efficacy and tumor localization between a masked and unmasked antibody. Overall, our simulations show that the efficacy induced by pacmilimab therapy is predicted to be similar to the unmasked version. The model also suggests that masking improves localization of the drug to the tumor. We also have shown how the key biomarkers, such as immune cell densities, change between responders and non-responders to this therapy. Simulations suggest that a higher ratio Teff/Treg ratio increases the ORR. This result is expected as effector T cells (Teff) have antitumoral activity while the Tregs function as inhibitors of the immune response. Contrarily, a higher M1/M2 ratio is associated with lower ORR. We then focused on an important biomarker, the number of T cell clones, TCC, which is an indirect measure of the tumor mutational burden, TMB, to mimic the pan-cancer observation that an increase in the mutation leads to an increase in response [26].

There are some limitations to this study which could be overcome in future studies. Firstly, virtual clinical results are only exploratory as there is no published clinical trial data on Pb-Tx anti-PD-L1 monotherapy applied to NSCLC. Additionally, we recognize that our model does not yet include some important components of the TME. The model does not account for cancer-associated fibroblasts (CAFs), which have a pro-tumorigenic activity by secretion of pro-cancer cytokines, such as IFN-γ, which polarize and strengthen the cancer against anti-tumoral stimuli [27], growth factors, such as HGF [28], extracellular remodelling that inhibits T cell infiltration [29, 30]. Another limitation is that we have not extended the out-of-synapse compartment to other ligands besides PD-L1. This can readily be done by introducing the same synaptic kinetics for the out-of-synapse model. Additionally, the model presented in this paper only reproduces a single lesion and does not account for metastatic tumors. Recently, Arulraj et al. [31] have used omics data to extend a single lesion QSP model to include multiple metastases. This framework could be extended using the same methods with the only caveat of determining the correct cleavage rate in the metastases. We would also like to emphasize that our model does not include any emergent toxicities nor does it simulate specific organs; rather it lumps them all in a peripheral compartment. Therefore, the model could be expanded closer to a PBPK formulation with a toxicity component to further study the consequences of masking.

Overall, in this work we have extended a platform to describe the activation of cancer immunity cycle in response to a PD-L1 blocking Pb-Tx and we have demonstrated its predictive potential. Moreover, the framework allowed us to run an exploratory study on NSCLC and it has the potential to extend these results to pan-cancer applications.

## Electronic supplementary material

Below is the link to the electronic supplementary material.

Supplementary Material 1

Supplementary Material 2
