# Supplementary material for: Virtual clinical trials via a QSP immuno-oncology model to simulate the response to a conditionally activated PD-L1 targeting antibody in NSCLC
Source: J Pharmacokinet Pharmacodyn. 2024 Jun 10;51(6):747–57. doi: 10.1007/s10928-024-09928-5 (PMC11579200; doi:10.1007/s10928-024-09928-5)
Supplement: Supplementary file 1 — Supplementary Material 1 [file 10928_2024_9928_MOESM1_ESM.docx]

**Supplementary information**

**QSP-IO model formulation**

The QSP model is an adapted version of the model for NSCLC presented by Wang et al [1] where we have added the PROBODY^®^ activatable therapeutics (Pb-Tx) kinetics, described in the next section, as well as the out-of-synapse binding proposed in Ippolito et al. [2]. For a comprehensive view on the model, refer to [1], while here we only give an overview and describe the additional components.

The NSCLC model used in this work is a 4 compartment QSP-IO model composed of: the tumor, central, peripheral and tumor-draining lymph node (TDLN) compartments. The model is specific for single lesion tumors, while for metastases another compartment should be implemented, as done by [3]. The tumor compartment is where the cancer and stromal cells are found. Here the immune system, such as T cells and macrophages, interact with the tumor cells via the synaptic sub-compartments which includes key ligands, as mentioned in the manuscript. As done previously [2,4], we have assumed that the macrophages exist only in the M1 or M2 stage, though we recognize that the behavior is actually a spectrum. Additionally, the maturation of antigen presenting cells (APCs) occurs here due to the signals arising from the presence and death of the cancer cells. These mature APCs then can travel to the TDLN where antigen presentation for the maturation of T cells occurs. The mature T cells then can travel via the central compartment to the site of the tumor.

We assume intravenous (IV) infusion for the therapy, therefore the Pb-Tx dose delivered directly to the central compartment. Since the central compartment is connected to the other three, these molecules can diffuse to these compartments and bind to the target ligand.

**PROBODY^®^ therapeutics Molecule Dynamics**

We now proceed to describe in detail the Pb-Tx dynamics with which we have informed the model. As mentioned in the main text, a QSP model of the Pb-Tx dynamics was previously published in literature by Stroh et al. [5,6] and implemented in a QSP-IO formulation by [2]. In all compartments, the active states of the pacmilimab can interact with receptors, but both specific and nonspecific elimination of the bound masked antibodies do not occur in the central compartment. The kinetics of state change of the Pb-Tx are governed by two fixed parameters: the unmasking equilibrium constant, $K_{M}$, defined as the ratio between the unmasking and remasking rates, and the cleavage rate $k_{cvg}$, which represents the rate at which the proteases cleave the substrate connecting the mask to the antibody. The unmasking is stochastic and reversible while the cleaving is an irreversible removal of the mask. As in [2], we have assumed that the cleavage rate outside of the tumor is 1% that in the tumor. Additionally, since most of the previous transport kinetics of pacmilimab were set similar to those of another anti-PD-L1 immune-checkpoint inhibitor atezolizumab, we again assume that the masked antibodies have the same transport kinetics as atezolizumab. Following the assumption of Wang et al. [4] that the formation of the synapse between cells only occurs in the tumor and lymph node compartments, here we also explicitly model the out-of-synapse interactions between the Pb-Tx and the ligands only for cells present in these two compartments. We do want to emphasize that the out-of-synapse binding can occur even outside of these compartments, as multiple cells that are not involved in the immune-synaptic formation in these compartments could express PD-L1, such as fibroblasts in the periphery or Tcells traveling through the central compartment [4].

For the Pb-Tx module, we include the dynamics of the 6 free states as well as 9 bound states. The concentrations of the 6 free states are $P_{mm}$, $P_{mo}$, $P_{oo}$, $P_{cm}$, $P_{co}$ and $P_{cc}$, where there are 2 subscripts $m$, $o$ and $c$ to indicate the masked, open and cleaved state, respectively, of the active sites of the antibody, e.g. $P_{co}$ means that the mask of one site has been cleaved while the other mask is still present but is revealing the active site. The kinetics of the free conditional antibodies, for example, in the tumor compartment $T$ are:

| $\boldsymbol{V}_{\boldsymbol{T}}\frac{\boldsymbol{d}\left[ \boldsymbol{P}_{\boldsymbol{mm}} \right]_{\boldsymbol{T}}}{\boldsymbol{dt}}\boldsymbol{=-}\boldsymbol{Q}_{\boldsymbol{T}}\left( \frac{\left[ \boldsymbol{P}_{\boldsymbol{mm}} \right]_{\boldsymbol{T}}}{\boldsymbol{\gamma}_{\boldsymbol{T}}}\boldsymbol{-}\frac{\left[ \boldsymbol{P}_{\boldsymbol{mm}} \right]_{\boldsymbol{C}}}{\boldsymbol{\gamma}_{\boldsymbol{C}}} \right)\boldsymbol{-}\boldsymbol{q}_{\boldsymbol{LD}}\boldsymbol{V}_{\boldsymbol{T}}\frac{\left[ \boldsymbol{P}_{\boldsymbol{mm}} \right]_{\boldsymbol{T}}}{\boldsymbol{\gamma}_{\boldsymbol{T}}}\boldsymbol{-2}\boldsymbol{k}_{\boldsymbol{P,m}}\boldsymbol{V}_{\boldsymbol{T}}\left[ \boldsymbol{P}_{\boldsymbol{mm}} \right]_{\boldsymbol{T}}\boldsymbol{+}\frac{\boldsymbol{k}_{\boldsymbol{P,m}}}{\boldsymbol{K}_{\boldsymbol{M}}}\boldsymbol{V}_{\boldsymbol{T}}\left[ \boldsymbol{P}_{\boldsymbol{mo}} \right]_{\boldsymbol{T}}\boldsymbol{-2}\boldsymbol{k}_{\boldsymbol{cvg}}\boldsymbol{V}_{\boldsymbol{T}}\left[ \boldsymbol{P}_{\boldsymbol{mm}} \right]_{\boldsymbol{T}}\boldsymbol{,}$ | (S1) |
| --- | --- |
| $\boldsymbol{V}_{\boldsymbol{T}}\frac{\boldsymbol{d}\left[ \boldsymbol{P}_{\boldsymbol{mo}} \right]_{\boldsymbol{T}}}{\boldsymbol{dt}}\boldsymbol{=-}\boldsymbol{Q}_{\boldsymbol{T}}\left( \frac{\left[ \boldsymbol{P}_{\boldsymbol{mo}} \right]_{\boldsymbol{T}}}{\boldsymbol{\gamma}_{\boldsymbol{T}}}\boldsymbol{-}\frac{\left[ \boldsymbol{P}_{\boldsymbol{mo}} \right]_{\boldsymbol{C}}}{\boldsymbol{\gamma}_{\boldsymbol{C}}} \right)\boldsymbol{-}\boldsymbol{q}_{\boldsymbol{LD}}\boldsymbol{V}_{\boldsymbol{T}}\frac{\left[ \boldsymbol{P}_{\boldsymbol{mo}} \right]_{\boldsymbol{T}}}{\boldsymbol{\gamma}_{\boldsymbol{T}}}\boldsymbol{+2}\boldsymbol{k}_{\boldsymbol{P,m}}\boldsymbol{V}_{\boldsymbol{T}}\left[ \boldsymbol{P}_{\boldsymbol{mm}} \right]_{\boldsymbol{T}}\boldsymbol{-}\frac{\boldsymbol{k}_{\boldsymbol{P,m}}}{\boldsymbol{K}_{\boldsymbol{M}}}\boldsymbol{V}_{\boldsymbol{T}}\left[ \boldsymbol{P}_{\boldsymbol{mo}} \right]_{\boldsymbol{T}}\boldsymbol{-}\boldsymbol{k}_{\boldsymbol{P,m}}\boldsymbol{V}_{\boldsymbol{T}}\left[ \boldsymbol{P}_{\boldsymbol{mo}} \right]_{\boldsymbol{T}}\boldsymbol{+2}\frac{\boldsymbol{k}_{\boldsymbol{P,m}}}{\boldsymbol{K}_{\boldsymbol{M}}}\boldsymbol{V}_{\boldsymbol{T}}\left[ \boldsymbol{P}_{\boldsymbol{oo}} \right]_{\boldsymbol{T}}\boldsymbol{-2}\boldsymbol{k}_{\boldsymbol{cvg}}\boldsymbol{V}_{\boldsymbol{T}}\left[ \boldsymbol{P}_{\boldsymbol{mo}} \right]_{\boldsymbol{T}}\boldsymbol{,}$ | (S2) |
| $\boldsymbol{V}_{\boldsymbol{T}}\frac{\boldsymbol{d}\left[ \boldsymbol{P}_{\boldsymbol{oo}} \right]_{\boldsymbol{T}}}{\boldsymbol{dt}}\boldsymbol{=-}\boldsymbol{Q}_{\boldsymbol{T}}\left( \frac{\left[ \boldsymbol{P}_{\boldsymbol{oo}} \right]_{\boldsymbol{T}}}{\boldsymbol{\gamma}_{\boldsymbol{T}}}\boldsymbol{-}\frac{\left[ \boldsymbol{P}_{\boldsymbol{oo}} \right]_{\boldsymbol{C}}}{\boldsymbol{\gamma}_{\boldsymbol{C}}} \right)\boldsymbol{-}\boldsymbol{q}_{\boldsymbol{LD}}\boldsymbol{V}_{\boldsymbol{T}}\frac{\left[ \boldsymbol{P}_{\boldsymbol{oo}} \right]_{\boldsymbol{T}}}{\boldsymbol{\gamma}_{\boldsymbol{T}}}\boldsymbol{+}\boldsymbol{k}_{\boldsymbol{P,m}}\boldsymbol{V}_{\boldsymbol{T}}\left[ \boldsymbol{P}_{\boldsymbol{mo}} \right]_{\boldsymbol{T}}\boldsymbol{-2}\frac{\boldsymbol{k}_{\boldsymbol{P,m}}}{\boldsymbol{K}_{\boldsymbol{M}}}\boldsymbol{V}_{\boldsymbol{T}}\left[ \boldsymbol{P}_{\boldsymbol{oo}} \right]_{\boldsymbol{T}}\boldsymbol{-2}\boldsymbol{k}_{\boldsymbol{cvg}}\boldsymbol{V}_{\boldsymbol{T}}\left[ \boldsymbol{P}_{\boldsymbol{oo}} \right]_{\boldsymbol{T}}\boldsymbol{,}$ | (S3) |
| $\boldsymbol{V}_{\boldsymbol{T}}\frac{\boldsymbol{d}\left[ \boldsymbol{P}_{\boldsymbol{cm}} \right]_{\boldsymbol{T}}}{\boldsymbol{dt}}\boldsymbol{=-}\boldsymbol{Q}_{\boldsymbol{T}}\left( \frac{\left[ \boldsymbol{P}_{\boldsymbol{cm}} \right]_{\boldsymbol{T}}}{\boldsymbol{\gamma}_{\boldsymbol{T}}}\boldsymbol{-}\frac{\left[ \boldsymbol{P}_{\boldsymbol{cm}} \right]_{\boldsymbol{C}}}{\boldsymbol{\gamma}_{\boldsymbol{C}}} \right)\boldsymbol{-}\boldsymbol{q}_{\boldsymbol{LD}}\boldsymbol{V}_{\boldsymbol{T}}\frac{\left[ \boldsymbol{P}_{\boldsymbol{cm}} \right]_{\boldsymbol{T}}}{\boldsymbol{\gamma}_{\boldsymbol{T}}}\boldsymbol{-}\boldsymbol{k}_{\boldsymbol{P,m}}\boldsymbol{V}_{\boldsymbol{T}}\left[ \boldsymbol{P}_{\boldsymbol{cm}} \right]_{\boldsymbol{T}}\boldsymbol{+}\frac{\boldsymbol{k}_{\boldsymbol{P,m}}}{\boldsymbol{K}_{\boldsymbol{M}}}\boldsymbol{V}_{\boldsymbol{T}}\left[ \boldsymbol{P}_{\boldsymbol{co}} \right]_{\boldsymbol{T}}\boldsymbol{+2}\boldsymbol{k}_{\boldsymbol{cvg}}\boldsymbol{V}_{\boldsymbol{T}}\left[ \boldsymbol{P}_{\boldsymbol{mm}} \right]_{\boldsymbol{T}}\boldsymbol{+}\boldsymbol{k}_{\boldsymbol{cvg}}\boldsymbol{V}_{\boldsymbol{T}}\left[ \boldsymbol{P}_{\boldsymbol{mo}} \right]_{\boldsymbol{T}}\boldsymbol{-}\boldsymbol{k}_{\boldsymbol{cvg}}\boldsymbol{V}_{\boldsymbol{T}}\left[ \boldsymbol{P}_{\boldsymbol{cm}} \right]_{\boldsymbol{T}}\boldsymbol{,}$ | (S4) |
| $\boldsymbol{V}_{\boldsymbol{T}}\frac{\boldsymbol{d}\left[ \boldsymbol{P}_{\boldsymbol{co}} \right]_{\boldsymbol{T}}}{\boldsymbol{dt}}\boldsymbol{=-}\boldsymbol{Q}_{\boldsymbol{T}}\left( \frac{\left[ \boldsymbol{P}_{\boldsymbol{co}} \right]_{\boldsymbol{T}}}{\boldsymbol{\gamma}_{\boldsymbol{T}}}\boldsymbol{-}\frac{\left[ \boldsymbol{P}_{\boldsymbol{co}} \right]_{\boldsymbol{C}}}{\boldsymbol{\gamma}_{\boldsymbol{C}}} \right)\boldsymbol{-}\boldsymbol{q}_{\boldsymbol{LD}}\boldsymbol{V}_{\boldsymbol{T}}\frac{\left[ \boldsymbol{P}_{\boldsymbol{co}} \right]_{\boldsymbol{T}}}{\boldsymbol{\gamma}_{\boldsymbol{T}}}\boldsymbol{+}\boldsymbol{k}_{\boldsymbol{P,m}}\boldsymbol{V}_{\boldsymbol{T}}\left[ \boldsymbol{P}_{\boldsymbol{cm}} \right]_{\boldsymbol{T}}\boldsymbol{-}\frac{\boldsymbol{k}_{\boldsymbol{P,m}}}{\boldsymbol{K}_{\boldsymbol{M}}}\boldsymbol{V}_{\boldsymbol{T}}\left[ \boldsymbol{P}_{\boldsymbol{co}} \right]_{\boldsymbol{T}}\boldsymbol{+}\boldsymbol{k}_{\boldsymbol{cvg}}\boldsymbol{V}_{\boldsymbol{T}}\left[ \boldsymbol{P}_{\boldsymbol{mo}} \right]_{\boldsymbol{T}}\boldsymbol{+2}\boldsymbol{k}_{\boldsymbol{cvg}}\boldsymbol{V}_{\boldsymbol{T}}\left[ \boldsymbol{P}_{\boldsymbol{oo}} \right]_{\boldsymbol{T}}\boldsymbol{-}\boldsymbol{k}_{\boldsymbol{cvg}}\boldsymbol{V}_{\boldsymbol{T}}\left[ \boldsymbol{P}_{\boldsymbol{co}} \right]_{\boldsymbol{T}}\boldsymbol{,}$ | (S5) |
| $\boldsymbol{V}_{\boldsymbol{T}}\frac{\boldsymbol{d}\left[ \boldsymbol{P}_{\boldsymbol{cc}} \right]_{\boldsymbol{T}}}{\boldsymbol{dt}}\boldsymbol{=-}\boldsymbol{Q}_{\boldsymbol{T}}\left( \frac{\left[ \boldsymbol{P}_{\boldsymbol{cc}} \right]_{\boldsymbol{T}}}{\boldsymbol{\gamma}_{\boldsymbol{T}}}\boldsymbol{-}\frac{\left[ \boldsymbol{P}_{\boldsymbol{cc}} \right]_{\boldsymbol{C}}}{\boldsymbol{\gamma}_{\boldsymbol{C}}} \right)\boldsymbol{-}\boldsymbol{q}_{\boldsymbol{LD}}\boldsymbol{V}_{\boldsymbol{T}}\frac{\left[ \boldsymbol{P}_{\boldsymbol{cc}} \right]_{\boldsymbol{T}}}{\boldsymbol{\gamma}_{\boldsymbol{T}}}\boldsymbol{+}\boldsymbol{k}_{\boldsymbol{cvg}}\boldsymbol{V}_{\boldsymbol{T}}\left[ \boldsymbol{P}_{\boldsymbol{cm}} \right]_{\boldsymbol{T}}\boldsymbol{+}\boldsymbol{k}_{\boldsymbol{cvg}}\boldsymbol{V}_{\boldsymbol{T}}\left[ \boldsymbol{P}_{\boldsymbol{co}} \right]_{\boldsymbol{T}}\boldsymbol{,}$ | (S6) |

where $k_{P,m}$ is the unmasking rate, $K_{M}$ is the equilibrium constant between the masked and unmasked states and $k_{cvg}$ is the cleavage rate. The kinetics of the other compartments are similar, with the exception of the cleavage rate being only $1\%$ and that there is also an additional term for the clearance in the central compartment, i.e. $k_{cl}{[P_{cc}]}_{C}$. The 9 bound states of the Pb-Tx are the monovalently bound, $P_{mo,fb}$, $P_{oo,fb}$, $P_{cm,bf}$, $P_{co,bf}$, $P_{co,fb}$, $P_{cc,fb}$, and the bivalently bound states, $P_{oo,bb}$, $P_{co,bb}$ and $P_{cc,bb}$, where the subscripts $f$ and $b$ indicate which site is free and which is bound, respectively. For example, $P_{co,bf}$ means that the cleaved site is bound while the open one is free, which is different from $P_{co,fb}$, where instead the open site is bound and the cleaved site is free. The monovalently and bivalently bound states have dynamics both inside and out-of-synapse with the addition of the transition between states due to Pb-Tx unmasking and substrate cleaving.

| $\frac{\boldsymbol{d}\left[ \boldsymbol{P}_{\boldsymbol{mo,fb}} \right]}{\boldsymbol{dt}}\boldsymbol{=}\boldsymbol{k}_{\boldsymbol{on,PDL}\boldsymbol{1,aPDL}\boldsymbol{1}}\left[ \boldsymbol{PDL}\boldsymbol{1} \right]\frac{\left[ \boldsymbol{P}_{\boldsymbol{mo}} \right]_{\boldsymbol{T}}}{\boldsymbol{\gamma}_{\boldsymbol{T}}}\boldsymbol{-}\boldsymbol{k}_{\boldsymbol{off,PDL}\boldsymbol{1,aPDL}\boldsymbol{1}}\left[ \boldsymbol{P}_{\boldsymbol{mo,fb}} \right]\boldsymbol{-}\boldsymbol{k}_{\boldsymbol{P,m}}\left[ \boldsymbol{P}_{\boldsymbol{mo,fb}} \right]\boldsymbol{+}\frac{\boldsymbol{k}_{\boldsymbol{P,m}}}{\boldsymbol{K}_{\boldsymbol{M}}}\left[ \boldsymbol{P}_{\boldsymbol{oo,fb}} \right]\boldsymbol{-2}\boldsymbol{k}_{\boldsymbol{cvg}}\boldsymbol{[}\boldsymbol{P}_{\boldsymbol{mo,fb}}\boldsymbol{],}$ | (S7) |
| --- | --- |
| $\frac{\boldsymbol{d}\left[ \boldsymbol{P}_{\boldsymbol{oo,fb}} \right]}{\boldsymbol{dt}}\boldsymbol{=2}\boldsymbol{k}_{\boldsymbol{on,PDL}\boldsymbol{1,aPDL}\boldsymbol{1}}\left[ \boldsymbol{PDL}\boldsymbol{1} \right]\frac{\left[ \boldsymbol{P}_{\boldsymbol{oo}} \right]_{\boldsymbol{T}}}{\boldsymbol{\gamma}_{\boldsymbol{T}}}\boldsymbol{-}\boldsymbol{k}_{\boldsymbol{off,PDL}\boldsymbol{1,aPDL}\boldsymbol{1}}\left[ \boldsymbol{P}_{\boldsymbol{oo,fb}} \right]\boldsymbol{+}\boldsymbol{k}_{\boldsymbol{P,m}}\left[ \boldsymbol{P}_{\boldsymbol{mo,fb}} \right]\boldsymbol{-}\frac{\boldsymbol{k}_{\boldsymbol{P,m}}}{\boldsymbol{K}_{\boldsymbol{M}}}\left[ \boldsymbol{P}_{\boldsymbol{oo,fb}} \right]\boldsymbol{-2}\boldsymbol{k}_{\boldsymbol{cvg}}\left[ \boldsymbol{P}_{\boldsymbol{oo,fb}} \right]\boldsymbol{-}\boldsymbol{\chi}_{\boldsymbol{aPDL}\boldsymbol{1}}\frac{\boldsymbol{k}_{\boldsymbol{on,PDL}\boldsymbol{1,aPDL}\boldsymbol{1}}}{\boldsymbol{d}_{\boldsymbol{syn}}\boldsymbol{N}_{\boldsymbol{A}}}\left[ \boldsymbol{P}_{\boldsymbol{oo,fb}} \right]\left[ \boldsymbol{PDL}\boldsymbol{1} \right]\boldsymbol{+2}\boldsymbol{k}_{\boldsymbol{off,PDL}\boldsymbol{1,aPDL}\boldsymbol{1}}\left[ \boldsymbol{P}_{\boldsymbol{oo,bb}} \right]\boldsymbol{,}$ | (S8) |
| $\frac{\boldsymbol{d}\left[ \boldsymbol{P}_{\boldsymbol{cm,bf}} \right]}{\boldsymbol{dt}}\boldsymbol{=}\boldsymbol{k}_{\boldsymbol{on,PDL}\boldsymbol{1,aPDL}\boldsymbol{1}}\left[ \boldsymbol{PDL}\boldsymbol{1} \right]\frac{\left[ \boldsymbol{P}_{\boldsymbol{cm}} \right]_{\boldsymbol{T}}}{\boldsymbol{\gamma}_{\boldsymbol{T}}}\boldsymbol{-}\boldsymbol{k}_{\boldsymbol{off,PDL}\boldsymbol{1,aPDL}\boldsymbol{1}}\left[ \boldsymbol{P}_{\boldsymbol{cm,bf}} \right]\boldsymbol{-}\boldsymbol{k}_{\boldsymbol{P,m}}\left[ \boldsymbol{P}_{\boldsymbol{cm,bf}} \right]\boldsymbol{+}\frac{\boldsymbol{k}_{\boldsymbol{P,m}}}{\boldsymbol{K}_{\boldsymbol{M}}}\left[ \boldsymbol{P}_{\boldsymbol{co,bf}} \right]\boldsymbol{+}\boldsymbol{k}_{\boldsymbol{cvg}}\boldsymbol{[}\boldsymbol{P}_{\boldsymbol{mo,fb}}\boldsymbol{]-}\boldsymbol{k}_{\boldsymbol{cvg}}\boldsymbol{[}\boldsymbol{P}_{\boldsymbol{cm,bf}}\boldsymbol{],}$ | (S9) |
| $\frac{\boldsymbol{d}\left[ \boldsymbol{P}_{\boldsymbol{co,bf}} \right]}{\boldsymbol{dt}}\boldsymbol{=}\boldsymbol{k}_{\boldsymbol{on,PDL}\boldsymbol{1,aPDL}\boldsymbol{1}}\left[ \boldsymbol{PDL}\boldsymbol{1} \right]\frac{\left[ \boldsymbol{P}_{\boldsymbol{co}} \right]_{\boldsymbol{T}}}{\boldsymbol{\gamma}_{\boldsymbol{T}}}\boldsymbol{-}\boldsymbol{k}_{\boldsymbol{off,PDL}\boldsymbol{1,aPDL}\boldsymbol{1}}\left[ \boldsymbol{P}_{\boldsymbol{co,bf}} \right]\boldsymbol{+}\boldsymbol{k}_{\boldsymbol{P,m}}\left[ \boldsymbol{P}_{\boldsymbol{cm,bf}} \right]\boldsymbol{-}\frac{\boldsymbol{k}_{\boldsymbol{P,m}}}{\boldsymbol{K}_{\boldsymbol{M}}}\left[ \boldsymbol{P}_{\boldsymbol{co,bf}} \right]\boldsymbol{+}\boldsymbol{k}_{\boldsymbol{cvg}}\boldsymbol{[}\boldsymbol{P}_{\boldsymbol{oo,fb}}\boldsymbol{]-}\boldsymbol{k}_{\boldsymbol{cvg}}\boldsymbol{[}\boldsymbol{P}_{\boldsymbol{co,bf}}\boldsymbol{]-}\boldsymbol{\chi}_{\boldsymbol{aPDL}\boldsymbol{1}}\frac{\boldsymbol{k}_{\boldsymbol{on,PDL}\boldsymbol{1,aPDL}\boldsymbol{1}}}{\boldsymbol{d}_{\boldsymbol{syn}}\boldsymbol{N}_{\boldsymbol{A}}}\left[ \boldsymbol{P}_{\boldsymbol{co,bf}} \right]\left[ \boldsymbol{PDL}\boldsymbol{1} \right]\boldsymbol{+}\boldsymbol{k}_{\boldsymbol{off,PDL}\boldsymbol{1,aPDL}\boldsymbol{1}}\left[ \boldsymbol{P}_{\boldsymbol{co,bb}} \right]\boldsymbol{,}$ | (S10) |
| $\frac{\boldsymbol{d}\left[ \boldsymbol{P}_{\boldsymbol{co,fb}} \right]}{\boldsymbol{dt}}\boldsymbol{=}\boldsymbol{k}_{\boldsymbol{on,PDL}\boldsymbol{1,aPDL}\boldsymbol{1}}\left[ \boldsymbol{PDL}\boldsymbol{1} \right]\frac{\left[ \boldsymbol{P}_{\boldsymbol{co}} \right]_{\boldsymbol{T}}}{\boldsymbol{\gamma}_{\boldsymbol{T}}}\boldsymbol{-}\boldsymbol{k}_{\boldsymbol{off,PDL}\boldsymbol{1,aPDL}\boldsymbol{1}}\left[ \boldsymbol{P}_{\boldsymbol{co,fb}} \right]\boldsymbol{+}\boldsymbol{k}_{\boldsymbol{cvg}}\boldsymbol{[}\boldsymbol{P}_{\boldsymbol{oo,fb}}\boldsymbol{]+}\boldsymbol{k}_{\boldsymbol{cvg}}\boldsymbol{[}\boldsymbol{P}_{\boldsymbol{mo,fb}}\boldsymbol{]-}\boldsymbol{k}_{\boldsymbol{cvg}}\boldsymbol{[}\boldsymbol{P}_{\boldsymbol{co,bf}}\boldsymbol{]-}\boldsymbol{\chi}_{\boldsymbol{aPDL}\boldsymbol{1}}\frac{\boldsymbol{k}_{\boldsymbol{on,PDL}\boldsymbol{1,aPDL}\boldsymbol{1}}}{\boldsymbol{d}_{\boldsymbol{syn}}\boldsymbol{N}_{\boldsymbol{A}}}\left[ \boldsymbol{P}_{\boldsymbol{co,fb}} \right]\left[ \boldsymbol{PDL}\boldsymbol{1} \right]\boldsymbol{+}\boldsymbol{k}_{\boldsymbol{off,PDL}\boldsymbol{1,aPDL}\boldsymbol{1}}\left[ \boldsymbol{P}_{\boldsymbol{oo,bb}} \right]\boldsymbol{,}$ | (S11) |
| $\frac{\boldsymbol{d}\left[ \boldsymbol{P}_{\boldsymbol{cc,fb}} \right]}{\boldsymbol{dt}}\boldsymbol{=2}\boldsymbol{k}_{\boldsymbol{on,PDL}\boldsymbol{1,aPDL}\boldsymbol{1}}\left[ \boldsymbol{PDL}\boldsymbol{1} \right]\frac{\left[ \boldsymbol{P}_{\boldsymbol{cc}} \right]_{\boldsymbol{T}}}{\boldsymbol{\gamma}_{\boldsymbol{T}}}\boldsymbol{-}\boldsymbol{k}_{\boldsymbol{off,PDL}\boldsymbol{1,aPDL}\boldsymbol{1}}\left[ \boldsymbol{P}_{\boldsymbol{co,fb}} \right]\boldsymbol{+}\boldsymbol{k}_{\boldsymbol{cvg}}\boldsymbol{[}\boldsymbol{P}_{\boldsymbol{co,fb}}\boldsymbol{]+}\boldsymbol{k}_{\boldsymbol{cvg}}\boldsymbol{[}\boldsymbol{P}_{\boldsymbol{co,bf}}\boldsymbol{]+}\boldsymbol{k}_{\boldsymbol{cvg}}\boldsymbol{-}\boldsymbol{\chi}_{\boldsymbol{aPDL}\boldsymbol{1}}\frac{\boldsymbol{k}_{\boldsymbol{on,PDL}\boldsymbol{1,aPDL}\boldsymbol{1}}}{\boldsymbol{d}_{\boldsymbol{syn}}\boldsymbol{N}_{\boldsymbol{A}}}\left[ \boldsymbol{P}_{\boldsymbol{cc,fb}} \right]\left[ \boldsymbol{PDL}\boldsymbol{1} \right]\boldsymbol{+2}\boldsymbol{k}_{\boldsymbol{off,PDL}\boldsymbol{1,aPDL}\boldsymbol{1}}\left[ \boldsymbol{P}_{\boldsymbol{cc,bb}} \right]\boldsymbol{,}$ | (S12) |
| $\frac{\boldsymbol{d}\left[ \boldsymbol{P}_{\boldsymbol{oo,bb}} \right]}{\boldsymbol{dt}}\boldsymbol{=}\boldsymbol{\chi}_{\boldsymbol{aPDL}\boldsymbol{1}}\frac{\boldsymbol{k}_{\boldsymbol{on,PDL}\boldsymbol{1,aPDL}\boldsymbol{1}}}{\boldsymbol{d}_{\boldsymbol{syn}}\boldsymbol{N}_{\boldsymbol{A}}}\left[ \boldsymbol{P}_{\boldsymbol{oo,fb}} \right]\left[ \boldsymbol{PDL}\boldsymbol{1} \right]\boldsymbol{-2}\boldsymbol{k}_{\boldsymbol{off,PDL}\boldsymbol{1,aPDL}\boldsymbol{1}}\left[ \boldsymbol{P}_{\boldsymbol{oo,bb}} \right]\boldsymbol{-2}\boldsymbol{k}_{\boldsymbol{cvg}}\boldsymbol{[}\boldsymbol{P}_{\boldsymbol{oo,bb}}\boldsymbol{],}$ | (S13) |
| $\frac{\boldsymbol{d}\left[ \boldsymbol{P}_{\boldsymbol{co,bb}} \right]}{\boldsymbol{dt}}\boldsymbol{=}\boldsymbol{\chi}_{\boldsymbol{aPDL}\boldsymbol{1}}\frac{\boldsymbol{k}_{\boldsymbol{on,PDL}\boldsymbol{1,aPDL}\boldsymbol{1}}}{\boldsymbol{d}_{\boldsymbol{syn}}\boldsymbol{N}_{\boldsymbol{A}}}\left[ \boldsymbol{P}_{\boldsymbol{co,bf}} \right]\left[ \boldsymbol{PDL}\boldsymbol{1} \right]\boldsymbol{+}\boldsymbol{\chi}_{\boldsymbol{aPDL}\boldsymbol{1}}\frac{\boldsymbol{k}_{\boldsymbol{on,PDL}\boldsymbol{1,aPDL}\boldsymbol{1}}}{\boldsymbol{d}_{\boldsymbol{syn}}\boldsymbol{N}_{\boldsymbol{A}}}\left[ \boldsymbol{P}_{\boldsymbol{co,fb}} \right]\left[ \boldsymbol{PDL}\boldsymbol{1} \right]\boldsymbol{-2}\boldsymbol{k}_{\boldsymbol{off,PDL}\boldsymbol{1,aPDL}\boldsymbol{1}}\left[ \boldsymbol{P}_{\boldsymbol{co,bb}} \right]\boldsymbol{+2}\boldsymbol{k}_{\boldsymbol{cvg}}\left[ \boldsymbol{P}_{\boldsymbol{oo,bb}} \right]\boldsymbol{-}\boldsymbol{k}_{\boldsymbol{cvg}}\boldsymbol{[}\boldsymbol{P}_{\boldsymbol{co,bb}}\boldsymbol{],}$ | (S14) |
| $\frac{\boldsymbol{d}\left[ \boldsymbol{P}_{\boldsymbol{cc,bb}} \right]}{\boldsymbol{dt}}\boldsymbol{=}\boldsymbol{\chi}_{\boldsymbol{aPDL}\boldsymbol{1}}\frac{\boldsymbol{k}_{\boldsymbol{on,PDL}\boldsymbol{1,aPDL}\boldsymbol{1}}}{\boldsymbol{d}_{\boldsymbol{syn}}\boldsymbol{N}_{\boldsymbol{A}}}\left[ \boldsymbol{P}_{\boldsymbol{cc,fb}} \right]\left[ \boldsymbol{PDL}\boldsymbol{1} \right]\boldsymbol{-2}\boldsymbol{k}_{\boldsymbol{off,PDL}\boldsymbol{1,aPDL}\boldsymbol{1}}\left[ \boldsymbol{P}_{\boldsymbol{cc,bb}} \right]\boldsymbol{+}\boldsymbol{k}_{\boldsymbol{cvg}}\left[ \boldsymbol{P}_{\boldsymbol{co,bb}} \right]\boldsymbol{.}$ | (S15) |

We want to emphasize that these Eqs. S7-S15 are implemented for both the synapse and out-of- synapse compartments, where we have assumed that the interaction characteristic length inside and outside of the synapse is still $d_{syn}$ and all the other parameters also do not vary.

**Out of synapse module**

Here we describe the additional out of synapse compartments that we have added to the model. The modeled cells that express PD-L1 in this framework are Tregs, cancer, macrophages and antigen presenting cells. While we have assumed that the synaptic area is the same independently of the participating cells, the area of the out-of-synapse depends on the cell area in question $A_{cell}$. Therefore, we need to declare a different out-of-synapse compartment for each of these four cells of area $A_{cell}-A_{syn}$. We also want to emphasize that the kinetic parameters do not change, with the exception of the absence of the reaction between PD-L1 and PD-1, which can occur only in the synapse. Additionally, we assume that the interaction distance of the ligand is equal to the synaptic distance $d_{syn}$. Thus, the equations describing the out-of-synapse kinetics are:

| $\frac{\boldsymbol{d}\left[ \boldsymbol{PDL}\boldsymbol{1:aPDL}\boldsymbol{1} \right]}{\boldsymbol{dt}}\boldsymbol{=2}\boldsymbol{k}_{\boldsymbol{on,PDL}\boldsymbol{1,aPDL}\boldsymbol{1}}\left[ \boldsymbol{PDL}\boldsymbol{1} \right]\frac{\left[ \boldsymbol{aPDL}\boldsymbol{1} \right]_{\boldsymbol{T}}}{\boldsymbol{\gamma}_{\boldsymbol{T}}}\boldsymbol{-}\boldsymbol{k}_{\boldsymbol{off,PDL}\boldsymbol{1,aPDL}\boldsymbol{1}}\left[ \boldsymbol{PDL}\boldsymbol{1:aPDL}\boldsymbol{1} \right]\boldsymbol{-}\boldsymbol{\chi}_{\boldsymbol{aPDL}\boldsymbol{1}}\frac{\boldsymbol{k}_{\boldsymbol{on,PDL}\boldsymbol{1,aPDL}\boldsymbol{1}}}{\boldsymbol{d}_{\boldsymbol{syn}}\boldsymbol{N}_{\boldsymbol{A}}}\left[ \boldsymbol{PDL}\boldsymbol{1:aPDL}\boldsymbol{1} \right]\left[ \boldsymbol{PDL}\boldsymbol{1} \right]\boldsymbol{+2}\boldsymbol{k}_{\boldsymbol{off,PDL}\boldsymbol{1,aPDL}\boldsymbol{1}}\left[ \boldsymbol{PDL}\boldsymbol{1:aPDL}\boldsymbol{1:PDL}\boldsymbol{1} \right]\boldsymbol{,}$ | (S16) |
| --- | --- |
| $\frac{\boldsymbol{d}\left[ \boldsymbol{PDL}\boldsymbol{1:aPDL}\boldsymbol{1:PDL}\boldsymbol{1} \right]}{\boldsymbol{dt}}\boldsymbol{=}\boldsymbol{\chi}_{\boldsymbol{aPDL}\boldsymbol{1}}\frac{\boldsymbol{k}_{\boldsymbol{on,PDL}\boldsymbol{1,aPDL}\boldsymbol{1}}}{\boldsymbol{d}_{\boldsymbol{syn}}\boldsymbol{N}_{\boldsymbol{A}}}\left[ \boldsymbol{PDL}\boldsymbol{1:aPDL}\boldsymbol{1} \right]\left[ \boldsymbol{PDL}\boldsymbol{1} \right]\boldsymbol{-2}\boldsymbol{k}_{\boldsymbol{off,PDL}\boldsymbol{1,aPDL}\boldsymbol{1}}\left[ \boldsymbol{PDL}\boldsymbol{1:aPDL}\boldsymbol{1:PDL}\boldsymbol{1} \right]\boldsymbol{,}$ | (S17) |
| $\frac{\boldsymbol{d}\left[ \boldsymbol{PDL}\boldsymbol{1} \right]}{\boldsymbol{dt}}\boldsymbol{=}\frac{\boldsymbol{k}_{\boldsymbol{out,PDL}\boldsymbol{1}}}{\boldsymbol{A}_{\boldsymbol{cell}}\boldsymbol{-}\boldsymbol{A}_{\boldsymbol{syn}}}\frac{\left[ \boldsymbol{IFN\gamma} \right]}{\left[ \boldsymbol{IFN\gamma} \right]\boldsymbol{+IFN}\boldsymbol{\gamma}_{\boldsymbol{50}}}\left( \boldsymbol{1-}\frac{\left[ \boldsymbol{PDL}\boldsymbol{1} \right]_{\boldsymbol{total}}}{{\boldsymbol{r}_{\boldsymbol{PDL}\boldsymbol{1,IFN\gamma}}\left[ \boldsymbol{PDL}\boldsymbol{1} \right]}_{\boldsymbol{baseline}}} \right)\boldsymbol{+}\boldsymbol{k}_{\boldsymbol{in,PDL}\boldsymbol{1}}\left( \left[ \boldsymbol{PDL}\boldsymbol{1} \right]_{\boldsymbol{baseline}}\boldsymbol{-}\left[ \boldsymbol{PDL}\boldsymbol{1} \right]_{\boldsymbol{total}} \right)\boldsymbol{-2}\boldsymbol{k}_{\boldsymbol{on,PDL}\boldsymbol{1,aPDL}\boldsymbol{1}}\left[ \boldsymbol{PDL}\boldsymbol{1} \right]\frac{\left[ \boldsymbol{aPDL}\boldsymbol{1} \right]_{\boldsymbol{T}}}{\boldsymbol{\gamma}_{\boldsymbol{T}}}\boldsymbol{+}\boldsymbol{k}_{\boldsymbol{off,PDL}\boldsymbol{1,aPDL}\boldsymbol{1}}\left[ \boldsymbol{PDL}\boldsymbol{1:aPDL}\boldsymbol{1} \right]\boldsymbol{-}\boldsymbol{\chi}_{\boldsymbol{aPDL}\boldsymbol{1}}\frac{\boldsymbol{k}_{\boldsymbol{on,PDL}\boldsymbol{1,aPDL}\boldsymbol{1}}}{\boldsymbol{d}_{\boldsymbol{syn}}\boldsymbol{N}_{\boldsymbol{A}}}\left[ \boldsymbol{PDL}\boldsymbol{1:aPDL}\boldsymbol{1} \right]\left[ \boldsymbol{PDL}\boldsymbol{1} \right]\boldsymbol{+2}\boldsymbol{k}_{\boldsymbol{off,PDL}\boldsymbol{1,aPDL}\boldsymbol{1}}\left[ \boldsymbol{PDL}\boldsymbol{1:aPDL}\boldsymbol{1:PDL}\boldsymbol{1 )} \right]\boldsymbol{,}$ | (S18) |

While the model is physically more realistic, our simulations have demonstrated negligible effect of the addition of the out-of-synaptic compartments. There are two main reasons for this counterintuitive effect. In the main text, we have alluded to the absence of PD-L1 cellular pathways besides the immune checkpoint formation. Therefore, the bonds forming between the PD-L1 and Pb-Tx or antibody do not result in any pharmacodynamic behavior. Finally, the kinetics of the free Pb-Tx and antibody remain also unchanged, since we assume an abundance of Pb-Tx and antibody in each compartment compared to the ones interacting with the cell surface.

**Influence of each immune fraction on the virtual population selection**

In this work, we have tailored our virtual patient selected criteria by including real patient omics data provided by iAtlas, though we would note that there are limitations in this method since it is biopsy dependent. Our procedure was introduced in the manuscript and it is a modification of [1], which was originally introduced by [7] and we refer to the latter for additional mathematical information. The procedure follows the steps described below:

1. Generate the proposed patient distribution: here we randomly sample (using Latin Hypercube Sampling [8, 9]) from precalibrated distributions of patient specific parameters;
2. Select the plausible patients from the proposed patients: run pre-treatment simulations for 1000 days to determine which proposed patients lead to realistic initial conditions, as defined in [1]. Those that satisfy this condition are sampled as part of the proposed patient cohort;
3. Calculate the immune subset ratios, $x_{i}$, for both the proposed patient cohort and the data from the iAtlas data;
4. Generate probability distributions for the proposed patient cohort, $p_{s}\left( x_{i} \right)$, and iAtlas data, $p_{d}\left( x_{i} \right)$, from the relative immune subset ratios;
5. Define the probability of inclusion for each $p=\beta p_{d}\left( x_{i} \right)/p_{s}\left( x_{i} \right)$, where $\beta$ is the normalizing factor and it is calculated via simulated annealing (since finding $\beta$ reduces to an optimization problem);
6. For each plausible patient, extract the relative $x_{i}$ and $p\left( x_{i} \right)$. With that probability, include the patient in the virtual patient cohort.

As a distinction from [1], we redefined the immune subset ratios and introduced a bounding parameter $\varepsilon$ to ensure that the values did not diverge in either the positive or negative directions, which would allow the use of more data points from the iAtlas (since some of these values are very small or zero). However, we wanted to ensure that for the same omics data our virtual patient cohort did not differ significantly from the one which would be selected by [1]. Therefore, as shown in Fig. S.1, we took the same plausible patient cohort of N=1500, the same iAtlas data, and we compared the virtual patient cohorts from the two selection criteria. We found that the two output cohorts were not statistically different, $p>0.25$, even though the actual number of VPs selected differed slightly likely due to the inherent stochasticity of the selection criteria via the probability of inclusion [7].


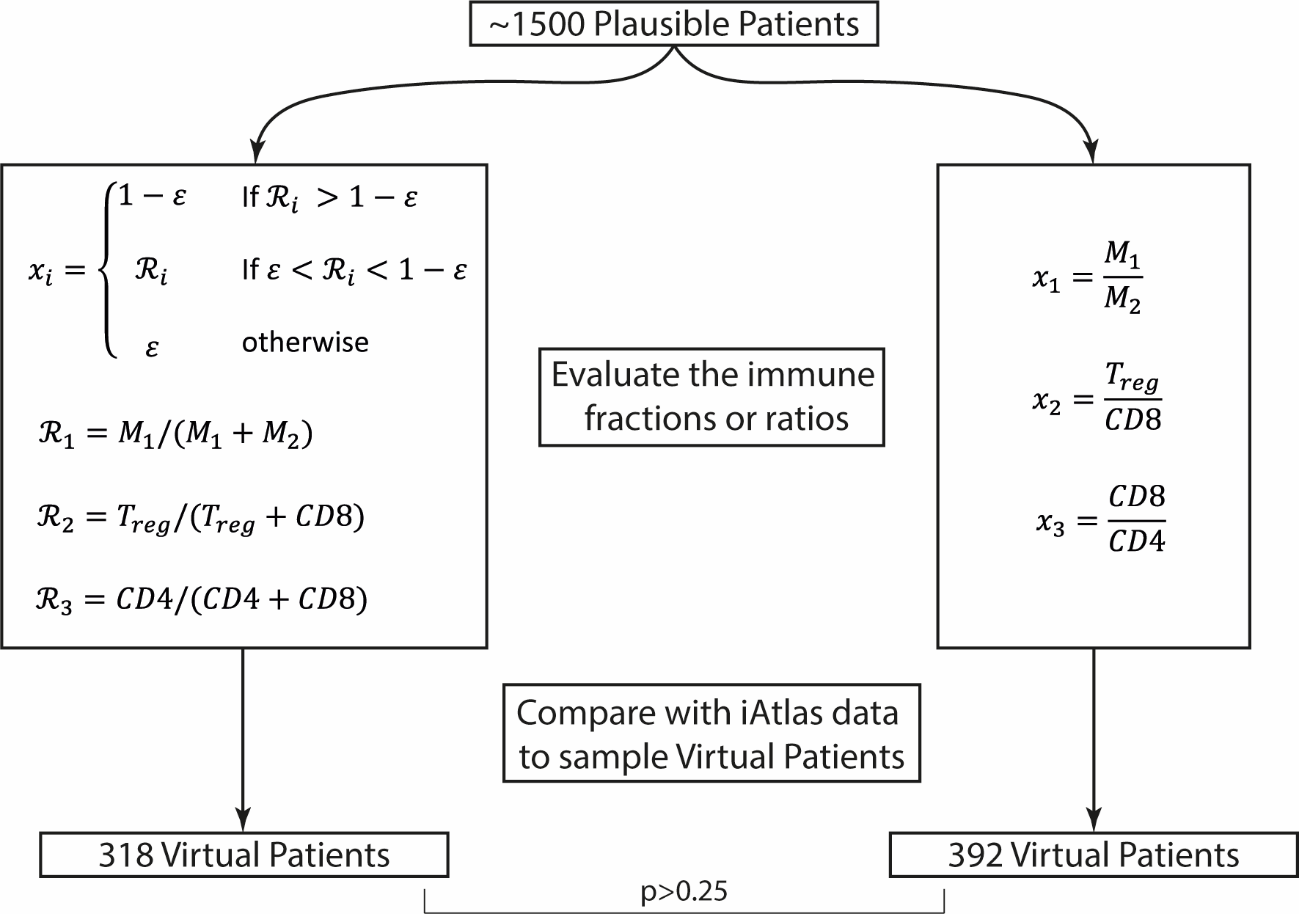


Figure S.1: Diagram showing the comparison between the VP cohort selected by previously published method [1] and the ratios used in this work.


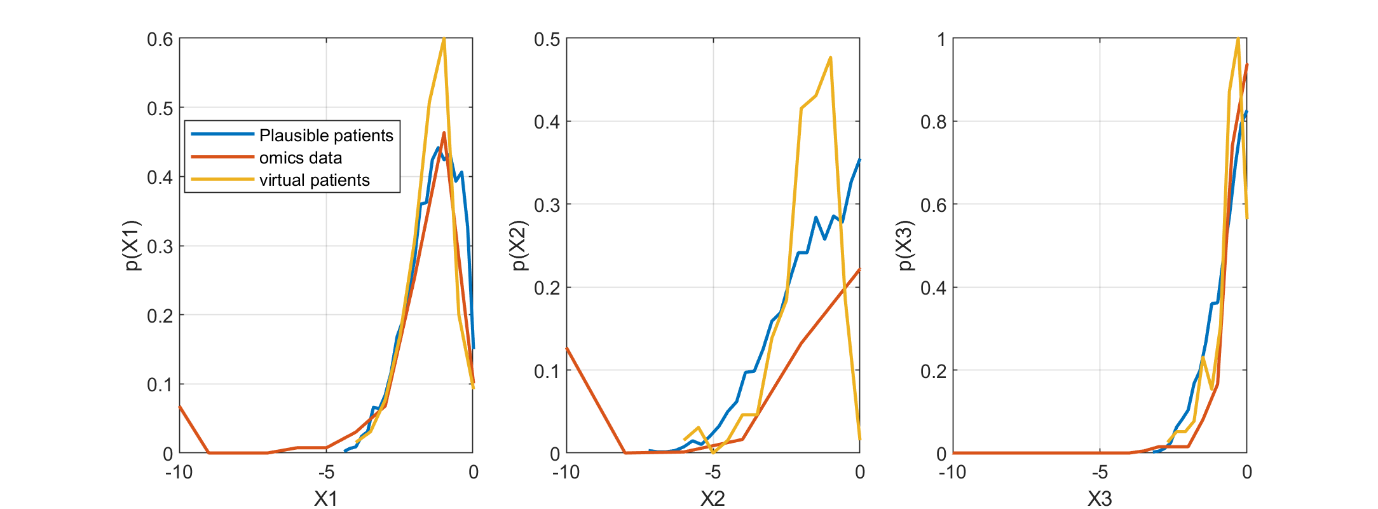


**Figure S.2**: Comparison between the plausible patient, real patient (omics data) and selected virtual patient probability density distribution of the immune ratios X1, X2 and X3 (see main text for expression of the immune ratios)..

The comparison between the plausible patient distribution, distribution from omics data and final virtual patient distribution is detailed in Fig S.2. While for X1 and X3 the virtual patient distribution is closer to the iAtlas data compared to the plausible patient distribution, there seems to be a discrepancy in X2. This is mainly due to the nature of the data: since a large number of points were low in value and thus set to $\varepsilon$, this resulted in a bimodal like distribution which overweighted points close to $\varepsilon$. While an apparent limitation of the method, it reflects its ability to include all the datapoints which in [1] would otherwise not be considered.

**Model implementation**

We implemented the entire model using the SimBiology toolbox from Matlab (MathWorks, Natick, MA). The numerical integration was done using the Sundials [10] solver already implemented in Matlab. Sundials uses several different packages for the adaptive time step integration of the system of differential equations. The timestep is selected as the largest integration interval where the integration error satisfies the constraints imposed by the absolute and relative tolerance. We select 1e-9 as the absolute tolerance and 1e-6 as the relative tolerance. The absolute tolerance represents the measure of the noise that we are willing to accept for the integration of our variables and here we fix the same value for all our integration variables. The results did not change significantly, i.e. below 1% difference, when these tolerances where varied within an order of magnitude. The integration data were stored for every simulated day rather than for each integration timestep.

The cleavage rate depends on the protease activity in the tumor microenvironment. We are aware that there is a large variability in the expression level which suggests variability in the activity level as well [11]. Therefore, we selected the distribution of $k_{cvg}$ as a uniform distribution, as done in [2], but we changed the mean to reflect the different protease activity in NSCLC compared to TNBC. The bounds situated at a 30% distance from the mean. We want to emphasize that future data on the enzymatic activity should definitely be used to better inform the model. Additionally, since the enzymatic activity is mostly in the tumor, similarly to Stroh et al. [4] we have assumed that the cleavage rate outside of the tumor is only 1% that inside the tumor compartment.

The dose of the unmasked antibody or Pb-Tx is administered in SimBiology as a dose object specified by dose amount, interval between doses and total number of doses. We assume that all the pacmilimab injected is initially in its full masked state, though we do recognize that it is possible that a percentage of it could be active due to the stochastic unmasking happening even before the injection. For the RECIST 1.1 [11] criteria, we evaluate the tumor size by assuming that the tumor is spherical in shape, as done in previous works [2,5,12,13].


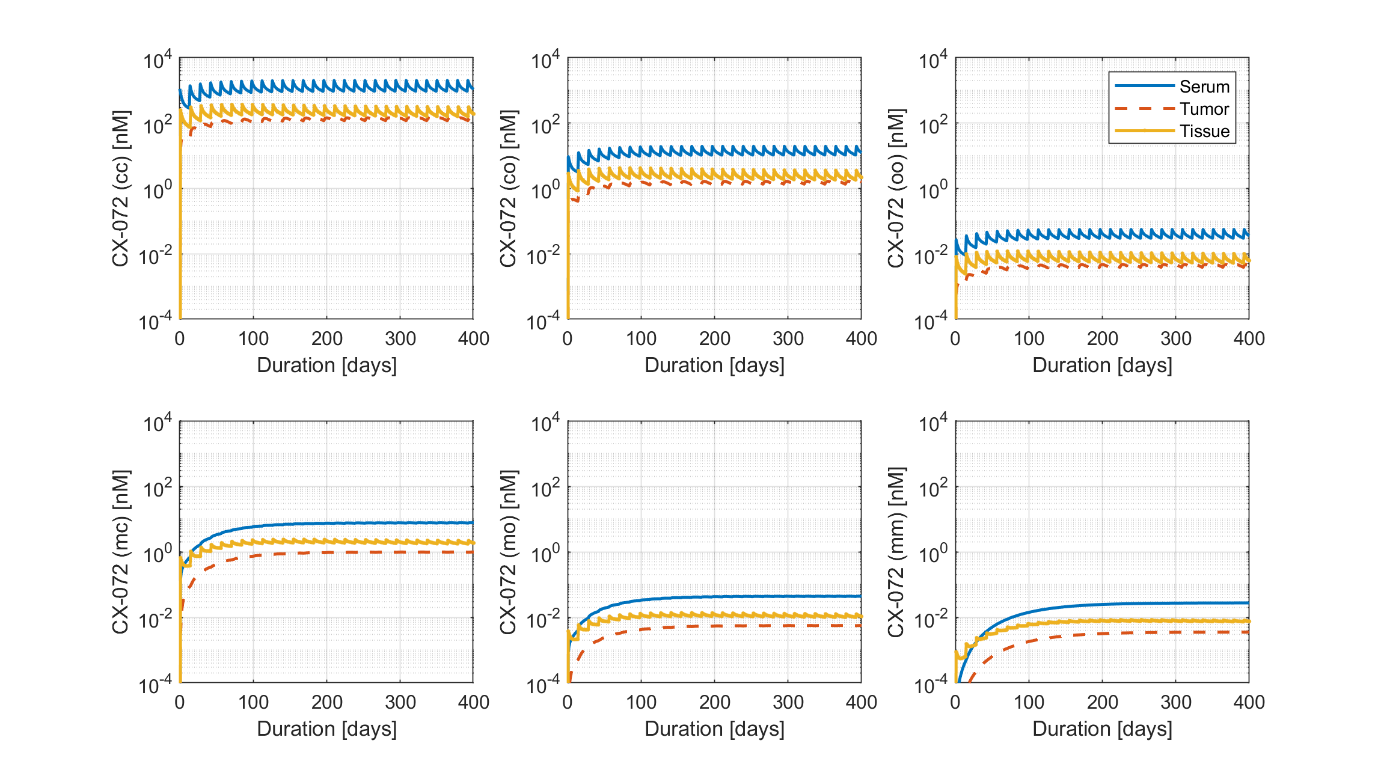


**Figure S.3**: PK of the different states of the PROBODY^®^ therapeutics molecule in serum (blue), tumor (red) and peripheral tissue (yellow).

**Additional statistical and technical methods**

Here we proceed to describe all the different statistical methods used to analyze the data in the various figures.

In Fig. 4b we use the p-value to measure the statistical difference between the distributions of each biomarker for responders and non-responders to the treatment. However, because the distributions are continuous and are not normal, we select the Wilcoxon test to measure the p-value. The global uncertainty and sensitivity analysis was performed using Partial Rank Correlation Coefficients methods.

Our model and simulations were run in Matlab 2023a, however, the procedure is extendable to any software that can run random sampling algorithms. The hardware requirements depend on the size of the VP cohort. With an 64bit CPU and 1 Tb SSD iMac we were easily able to simulate cohorts of more than 3000 plausible patients. The total run time for a cohort of 3000 plausible patients was around 3 hours. Each cohort is reproducible by initializing the random number generator to the same seed. Each virtual population is selected (and is reproducible) via the initiation of the random number generator to a fixed seed. While the patients of a given population are different from the next, the population distribution of parameters and variables will match between different but large enough cohorts. Note that the average time of selection of 3000 plausible patients and the respective VP cohort is around 2 hours.

We have developed the model using SimBiology Toolbox in Matlab, but it can be implemented in any software capable of solving ordinary and algebraic differential equations. SimBiology can export the model as SBML code but the compatibility with different a different platform is unknown.

**References**

1. Wang H, Arulraj T, Kimko H, Popel AS. (2023) Generating immunogenomic data-guided virtual patients using a QSP model to predict response of advanced NSCLC to PD-L1 inhibition. npj Precision Oncology.;7(1):55
2. Ippolito A, Wang H, Zhang Y, Vakil V, Bazzazi H, Popel AS. Eliciting the antitumor immune response with a conditionally activated PD‐L1 targeting antibody analyzed with a quantitative systems pharmacology model. CPT: Pharmacometrics & Systems Pharmacology. 2024 Jan;13(1):93-105.
3. Arulraj T, Wang H, Emens LA, Santa-Maria CA, Popel AS. (2023) Science Advances 2023 A transcriptome-informed QSP model of metastatic triple-negative breast cancer identifies predictive biomarkers for PD-1 inhibition. Sci Adv.;9(26):eadg0289.
4. Wang H, Zhao C, Santa-Maria CA, Emens LA, Popel AS. (2022) Dynamics of tumor-associated macrophages in a quantitative systems pharmacology model of immunotherapy in triple-negative breast cancer. iScience.;25(8):104702
5. Stroh M, Sagert J, Burke JM, Apgar JF, Lin L, Millard BL, Kavanaugh WM. (2019) Quantitative systems pharmacology model of a masked, tumor‐activated antibody. CPT: pharmacometrics & systems pharmacology.;8(9):676-84.
6. Stroh M, Green M, Millard BL, Apgar JF, Burke JM, Garner W, Lu H, Lyman SK, Desnoyers LR, Richardson J, Hannah A. (2021) Model‐Informed Drug Development of the Masked Anti‑PD‑L1 Antibody CX‐072. Clinical Pharmacology & Therapeutics.;109(2):383-93.
7. R. Allen, T. Rieger, C. Musante, (2016) Efficient Generation and Selection of Virtual Populations in Quantitative Systems Pharmacology Models. CPT Pharmacometrics Syst. Pharmacol. 5, 140–146.
8. Cheng Y, Straube R, Alnaif AE, Huang L, Leil TA, Schmidt BJ. (2022) Virtual populations for quantitative systems pharmacology models. In: Systems Medicine (pp. 129-179). New York, NY: Springer US.
9. Saltelli, A.; Ratto, M.; Andres, T.; Campolongo, F.; Cariboni, J.; Gatelli, D.; Saisana, M.; Tarantola, S. Global Sensitivity Analysis: The Primer; John Wiley & Sons: Hoboken, NJ, USA, 2008. pp. 10-99.
10. Hindmarsh AC, Brown PN, Grant KE, Lee SL, Serban R, Shumaker DE, Woodward CS. (2005) SUNDIALS: Suite of nonlinear and differential/algebraic equation solvers. ACM Transactions on Mathematical Software (TOMS).;31(3):363-96.
11. Eisenhauer EA, Therasse P, Bogaerts J, Schwartz LH, Sargent D, Ford R, Dancey J, Arbuck S, Gwyther S, Mooney M, Rubinstein L. (2009) New response evaluation criteria in solid tumours: revised RECIST guideline (version 1.1). European journal of cancer.;45(2):228-47.
12. Jafarnejad M, Gong C, Gabrielson E, Bartelink IH, Vicini P, Wang B, Narwal R, Roskos L, Popel AS. (2019) A computational model of neoadjuvant PD-1 inhibition in non-small cell lung cancer. The AAPS journal.;21(5):1-4.
13. Wang H, Ma H, Sové RJ, Emens LA, Popel AS. (2021) Quantitative systems pharmacology model predictions for efficacy of atezolizumab and nab-paclitaxel in triple-negative breast cancer. Journal for immunotherapy of cancer.;9(2).
